# Age- and stress-associated *C*. *elegans* granulins impair lysosomal function and induce a compensatory HLH-30/TFEB transcriptional response

**DOI:** 10.1371/journal.pgen.1008295

**Published:** 2019-08-09

**Authors:** Victoria J. Butler, Fuying Gao, Christian I. Corrales, Wilian A. Cortopassi, Benjamin Caballero, Mihir Vohra, Kaveh Ashrafi, Ana Maria Cuervo, Matthew P. Jacobson, Giovanni Coppola, Aimee W. Kao

**Affiliations:** 1 Memory and Aging Center, Department of Neurology, University of California, San Francisco, California, United States of America; 2 Semel Institute for Neuroscience and Human Behavior, Departments of Psychiatry and Biobehavioral Sciences and Neurology, David Geffen School of Medicine, University of California Los Angeles, Los Angeles, California, United States of America; 3 Department of Pharmaceutical Chemistry, University of California, San Francisco, California, United States of America; 4 Department of Developmental and Molecular Biology, Albert Einstein College of Medicine, New York, New York, United States of America; 5 Department of Physiology, University of California, San Francisco, California, United States of America; Veterans Affairs Puget Sound Health Care System, UNITED STATES

## Abstract

The progressive failure of protein homeostasis is a hallmark of aging and a common feature in neurodegenerative disease. As the enzymes executing the final stages of autophagy, lysosomal proteases are key contributors to the maintenance of protein homeostasis with age. We previously reported that expression of granulin peptides, the cleavage products of the neurodegenerative disease protein progranulin, enhance the accumulation and toxicity of TAR DNA binding protein 43 (TDP-43) in *Caenorhabditis elegans* (*C*. *elegans*). In this study we show that *C*. *elegans* granulins are produced in an age- and stress-dependent manner. Granulins localize to the endolysosomal compartment where they impair lysosomal protease expression and activity. Consequently, protein homeostasis is disrupted, promoting the nuclear translocation of the lysosomal transcription factor HLH-30/TFEB, and prompting cells to activate a compensatory transcriptional program. The three *C*. *elegans* granulin peptides exhibited distinct but overlapping functional effects in our assays, which may be due to amino acid composition that results in distinct electrostatic and hydrophobicity profiles. Our results support a model in which granulin production modulates a critical transition between the normal, physiological regulation of protease activity and the impairment of lysosomal function that can occur with age and disease.

## Author summary

Progressive decline in maintenance of protein homeostasis clearly contributes to the development of neurodegenerative disorders, yet the molecular basis of this decline is poorly understood. Here, we take advantage of molecular genetic techniques available in the model organism *C*. *elegans* to investigate the mechanism underlying neurodegenerative disease due to mutations in the progranulin gene. We find that age, gene mutation and physiological stress lead to the accumulation of lysosomal granulins (the cleavage products of the progranulin protein) thereby disrupting cellular protein homeostasis. Granulin expression impairs animal fitness, resistance to stress and neuronal function, and stimulates a lysosomal stress response in an attempt to up-regulate lysosomal genes and restore normal function. Our findings are particularly important because they suggest a new, rational target—inhibition of progranulin cleavage into granulins—for neurodegenerative disease therapy.

## Introduction

Aging and stress are thought to enhance neurodegenerative disease risk through the accumulation of misfolded and aggregated proteins [1–3]. The lysosome is the key degradative organelle within the cell [4], and therefore plays a pivotal role in the maintenance of protein homeostasis. It contains specialized enzymes, called cathepsins, which work optimally at the acidic pH in this compartment and have a crucial role in processing and degrading proteins [5]. The transcription factor EB (TFEB) controls the expression of genes involved in lysosomal biogenesis and function [6, 7]. TFEB dysregulation has been associated with neurodegenerative disease [8, 9] and its overexpression may help to promote the clearance of protein aggregates [10, 11]. Although, genetic and functional studies have implicated lysosomal dysfunction in the pathogenesis of multiple neurodegenerative diseases [12–14], understanding of the molecular basis of this phenomenon remains incomplete.

Heterozygous progranulin *(PGRN)* loss-of-function mutations lead to autosomal dominant transmission of the neurodegenerative disorder frontotemporal lobar degeneration (FTLD) with TAR DNA binding protein 43 (TDP-43) inclusions [15–17]. The molecular function of the progranulin protein (PGRN) remained elusive until it was indelibly linked to lysosomal function by the finding that loss of both gene alleles results in the lysosomal storage disease, neuronal ceroid lipofuscinosis [18]. Progranulin localizes to lysosomes [19–22] where it may act to promote lysosomal biogenesis and function [20, 23–25].

The progranulin (PGRN) protein can be proteolytically cleaved to liberate multiple cysteine-rich “granulin” peptides [26]. Granulins are highly conserved, disulfide-bonded miniproteins with unknown biological function [27–31]. Like progranulin, granulin peptides have been shown to localize to the endolysosomal compartment [32], and can be generated through the action of cysteine proteases on progranulin [32–34]. Owing to the twelve cysteines and six disulfide bonds found in each cleaved granulin, these peptides adopt a stacked β-sheet configuration that is compact, structurally stable and potentially protease resistant [35]. Several lines of evidence exist that cleaved granulin peptides oppose the function of the full-length protein. While progranulin has proliferative [35, 36] and anti-inflammatory [37, 38] properties, granulin peptides have been shown to inhibit cell growth [35] and stimulate inflammation [38]. In addition, we have previously demonstrated a role for *C*. *elegans* granulins in selectively promoting the accumulation of TDP-43, thereby exacerbating TDP-43 toxicity and potentially contributing to the pathogenesis of disease [39]. However, the mechanism by which granulins exert this specific regulation on TDP-43 metabolism remains unknown. *C*. *elegans* provides many advantages as a model system to study granulin function, including conservation of the progranulin gene, and the many available molecular and cell biology techniques.

In this study, we further investigate the molecular mechanisms of *C*. *elegans* granulins on lysosomal function and protein homeostasis. We show that *C*. *elegans* granulins localize to the endolysosomal fraction. Granulin production increases with age and stress, and granulin expression reduces animal fitness by impairing lysosomal protease expression and activity. This prompts cells to activate a compensatory transcriptional program involving HLH-30/TFEB nuclear translocation and up-regulation of the transcription of HLH-30/TFEB-related genes. Overall, our findings highlight granulins as critical regulators of proteolytic lysosomal function and potential drivers of neurodegenerative disease pathogenesis.

## Results

### *C*. *elegans* granulins impair organismal fitness and resistance to ER stress

**Fig 1:**
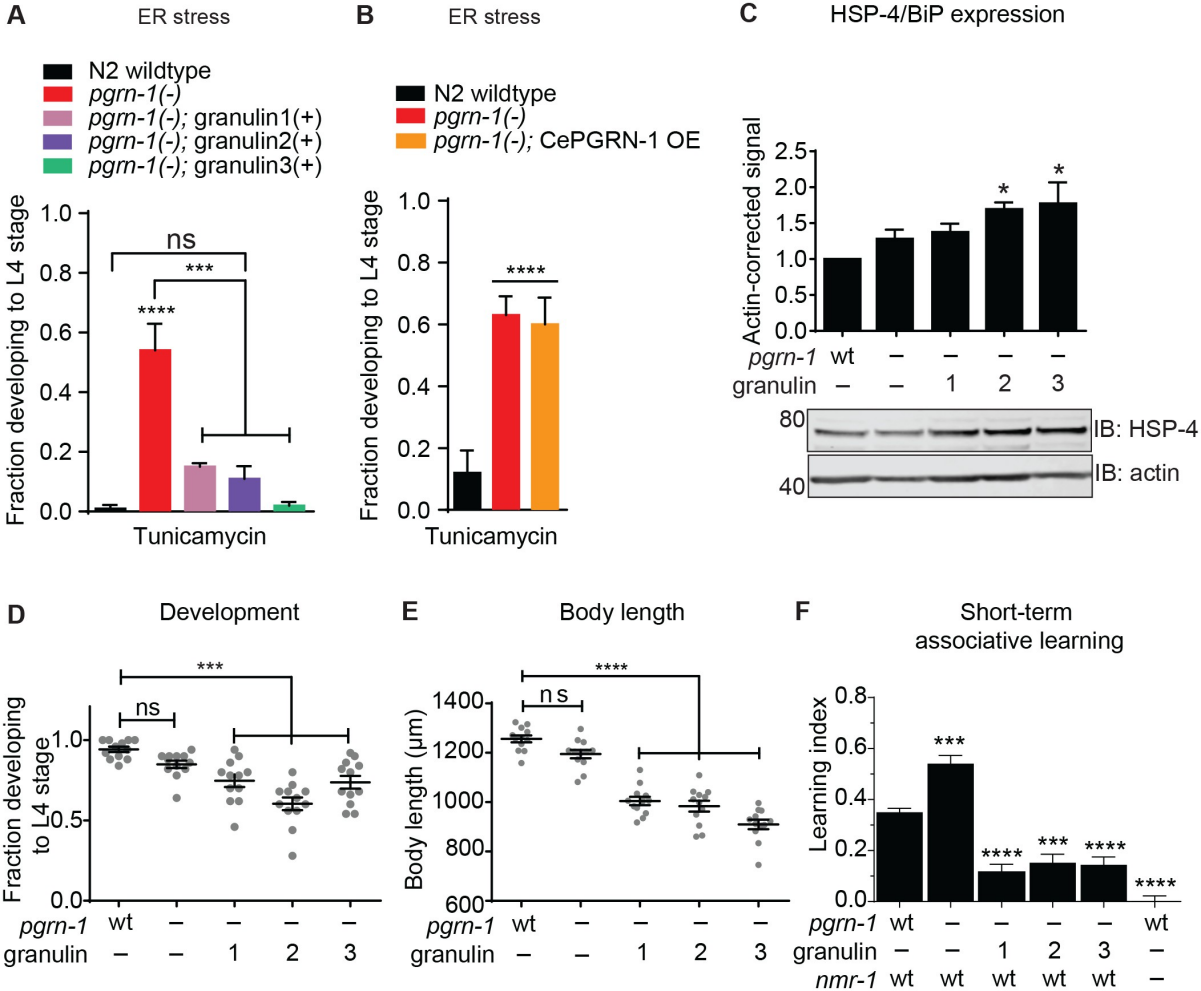
*C*. *elegans* granulins impair organismal fitness and resistance to ER stress. (**A**) Wild-type (N2) and *pgrn-1(-)* animals with and without granulin expression were subjected to ER stress with tunicamycin (5 μg / ml). The fraction developing to L4 stage was quantified (n = 50, 3 biological replicates). (**B**) Wild-type (N2) and *pgrn-1(-)* animals with and without *C*. *elegans* progranulin over-expression (OE) were subjected to ER stress with tunicamycin (5 μg / ml). The fraction developing to L4 stage was quantified (n = 50, 3 biological replicates). (**C**) Total worm lysates from synchronized day 1 adult granulin-expressing animals were immunoblotted with an anti-HSP-4/BiP antibody (3 biological replicates). Anti-actin was used as a loading control. (**D**) Wild-type and *pgrn-1(-)* animals with and without granulin expression were staged as embryos. Animals were scored for development to L4 stage (n = 50, 12 biological replicates). (**E**) Measurement of body length at day 1 adulthood (n = 12). (**F**) Measurement of short-term associative learning (three biological replicates). The glutamate receptor mutant *nmr-1(ak4)* was used as a positive control. Throughout, error bars show mean ± SEM, one or two-way ANOVA with post-hoc Tukey multiple comparisons test. Comparisons are to wild-type unless otherwise indicated (*P<0.05, ***P<0.001, ****P<0.0001, ns = not significant, wt = wild-type).

We have previously shown that *C*. *elegans* progranulin (*pgrn-1)* null mutants exhibit enhanced resistance to endoplasmic reticulum (ER) unfolded protein stress [40]. As a genetic null, *pgrn-1(-)* animals produce neither full-length progranulin nor cleaved granulins; therefore, absence of either the holoprotein or the cleavage fragments could be responsible for the ER stress resistance. Based on our earlier finding that granulins could exacerbate TDP-43 toxicity [39], we hypothesized that the bioactive granulins were responsible for inhibiting ER stress resistance. Hence, to isolate granulin activity, we expressed individual *C*. *elegans* granulins 1, 2 and 3 at comparable levels in a *pgrn-1* null background [39]. Granulin expression in a progranulin null background completely abolished the ER stress resistance phenotype (Fig 1A). In contrast, animals over-expressing *C*. *elegans* full-length progranulin in a progranulin null background remained ER stress resistant (Fig 1B). Over-expressed full-length progranulin was not cleaved under ER stress (S1A–S1D Fig), and could promote ER stress resistance in the presence of granulin (S1E Fig). Furthermore, transgenic expression of human tau protein and TDP-43 in a progranulin null background did not abrogate ER stress resistance (S1F Fig). Taken together, these data suggest that it is the granulins, and not full-length progranulin, that specifically inhibit ER stress resistance.

Given that granulins impair ER stress resistance, we wondered if they might more broadly impact protein homeostasis. Thus, we measured endogenous levels of heat shock protein HSP-4, the nematode homolog of human BiP/Grp78 [41]. HSP-4/BiP expression is upregulated during the unfolded protein response (UPR) [42]. We found that granulin-expressing animals displayed a trend for increased basal expression of HSP-4/BiP on day 1 of adulthood, reaching significance in animals expressing granulin 2 and 3 (Fig 1C). Therefore, in the absence of the progranulin holoprotein, granulin expression upregulates HSP-4 and this is indicative of UPR induction and perturbed protein homeostasis.

While working with the granulin-expressing lines, we noted a decrease in overall animal fitness attributable to the granulins. Granulin production significantly reduced animal viability by lowering the number of eggs that hatched and slowing the development of animals to maturity (Fig 1D). Granulin-expressing animals that did reach adulthood were smaller in size (Fig 1E). Short-term associative learning can be assayed in *C*. *elegans* using a positive olfactory learning paradigm [43, 44]. When granulin-expressing animals were tested in this assay they underperformed compared to controls (Fig 1F), suggesting that granulin expression may result in neuronal dysfunction. These data, coupled with previous work by others on the function of progranulin [35–38], suggest that granulins impair animal fitness, resistance to stress and neuronal function, while progranulin promotes these qualities.

### Granulins localize to the endolysosomal compartment

**Fig 2:**
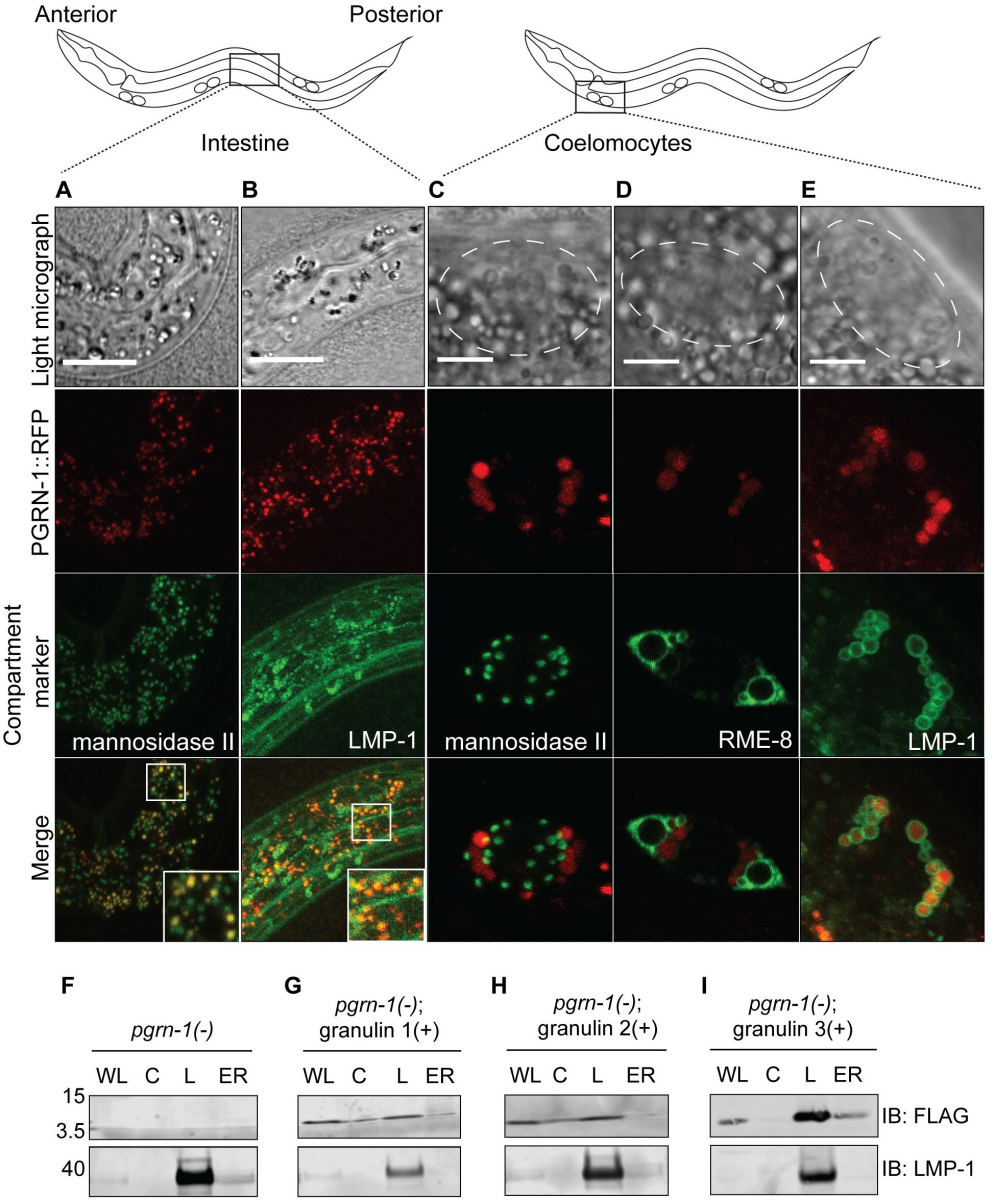
*C*. *elegans* progranulin and granulins localize to the endolysosomal compartment. A translational PGRN-1::RFP reporter (red) co-localizes with (**A**) Golgi (mannosidase II::GFP) and (**B**) lysosomes (LMP-1::GFP) in the intestine of L1 stage larvae (anterior to the left). In scavenging coelomocytes, PGRN-1::RFP does not co-localize with (**C**) Golgi (mannosidase II::GFP), but does co-localize with (**D**) early, late and recycling endosomes (RME-8::GFP) and (**E**) lysosomes (LMP-1::GFP) (n = 8 animals per GFP marker). Dashed white lines mark the outline of each coelomocyte cell and white boxes show zoomed insets in Fig 2A-B, scale bar = 5 μm. Shown are representative images from confocal microscopy z-stack sections taken at 0.7 μm. (**F-I**) Subcellular fractionation of *pgrn-1(-)* animals (**F**), and *pgrn-1(-)* animals with expression of (**G**) granulin 1, (**H**) granulin 2, and (**I**) granulin 3. Whole lysate (WL), cytosol (C), lysosome (L) and endoplasmic reticulum (ER) fractions (10 μg total protein) were immunoblotted with anti-FLAG and anti-LMP-1 antibodies.

To establish the trafficking and localization of granulin peptides within a whole organism, we utilized microscopy and biochemistry techniques. First, we determined the sub-cellular localization of full-length progranulin using a translational progranulin reporter, PGRN-1::RFP, and organelle-specific markers. As expected, in cells that secrete progranulin, such as the intestine, the reporter co-localized with both a Golgi marker, mannosidase II (Fig 2A), and a lysosomal marker, lysosomal-associated membrane protein 1 (LMP-1) (Fig 2B). However, in coelomocytes, a cell type that takes up but does not produce progranulin [45], the progranulin reporter was only seen in the endolysosomal compartment (Fig 2C–2E), suggesting that extracellular progranulin is transported through endosomes to reach the lysosome.

Having established that progranulin can be trafficked from one tissue type to another, we next sought to better understand the subcellular localization of granulin peptides. To do so, we developed a protocol for subcellular fractionation of *C*. *elegans*. The purity of cytosolic, ER and endolysosomal fractions was confirmed with established markers (S2 Fig). Individual granulins that were transgenically expressed also demonstrated lysosomal localization (Fig 2F–2I). Therefore, *C*. *elegans* progranulin and granulins localize to the endolysosomal compartment.

### Granulins are produced in an age- and stress-dependent manner and disrupt lysosomal morphology

**Fig 3:**
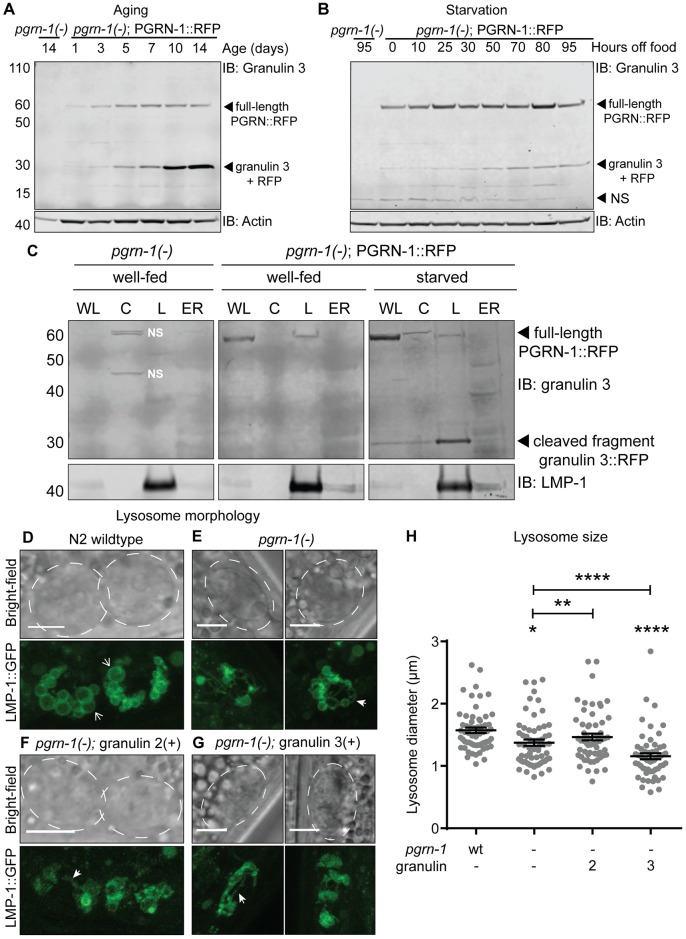
Granulins are produced in an age- and stress-dependent manner and disrupt lysosomal morphology. (**A-B**) Western blot of *C*. *elegans* PGRN-1::RFP lysates with (**A**) aging and (**B**) starvation. Immunoblotting was performed with an anti-granulin 3 antibody. NS = non-specific band. The most prominent cleavage product at ~30kDa was recognized by both granulin 3 and RFP antibodies (see S3E Fig). (**C**) Subcellular fractionation of *pgrn-1(-)*; PGRN::RFP animals. Whole lysate (WL), cytosol (C), lysosome (L) and endoplasmic reticulum (ER) fractions from fed and starved (70 hours off-food) animals were immunoblotted with anti-granulin 3 and anti-LMP-1 antibodies. The same progranulin full-length and cleavage bands were also identified with an anti-RFP antibody (S3F Fig). Well-fed *pgrn-1(-)* animals are shown as a control for non-specific bands (NS). (**D-G**) Representative light and fluorescent confocal images of anterior coelomocyte cells expressing LMP-1::GFP in (**D**) wild-type, (**E**) *pgrn-1(-)*, (**F**) *pgrn-1(-)*; granulin 2(+) and (**G**) *pgrn-1(-)*; granulin 3(+) animals. Animals were imaged at L4 stage. Scale bars are 10 μm in the wild-type panel and 5 μm in remaining panels. Dashed white lines mark the outline of each coelomocyte cell. Open white arrow heads indicate spherical lysosomes and closed white arrow heads indicate tubular extensions (number of animals with tubular extensions: wt: 2/22, *pgrn-1(-)*: 10/21, *pgrn-1(-)*; granulin 2(+): 7/25, *pgrn-1(-)*; granulin 3(+); 13/24). (**H**) Lysosomal diameter measurements from anterior coelomocyte cells of L4 stage animals (n = 60). Error bars show mean ± SEM, one-way ANOVA with post-hoc Tukey multiple comparisons test (mean values (μm): wt: 1.57 ± 0.04, *pgrn-1(-)*: 1.37 ± 0.05, *pgrn-1(-)*; granulin 2(+): 1.47 ± 0.05, *pgrn-1(-)*; granulin 3(+): 1.16 ± 0.05). Comparisons are to wild-type unless otherwise indicated (*P<0.05, **P<0.01, ****P<0.0001, wt = wild-type).

In *C*. *elegans* and mammals, progranulin production increases with age [45, 46] and injury [47, 48]. However, the degree to which granulin peptides are liberated has not been measured. We first asked if progranulin cleavage into granulins increases with age. Using our PGRN-1::RFP translational reporter, we found that granulin production does indeed increase in an age-dependent fashion (Fig 3A and S3A and S3B Fig), suggesting that either an increase in expression and cleavage of progranulin, and/or an age-associated decline in granulin turnover, contributes to granulin accumulation. Granulin cleavage also increased in response to certain physiological stressors such as starvation (Fig 3B and S3C and S3D Fig). Thus, age and stressful stimuli, such as starvation, appear to promote the cleavage of full-length progranulin into granulins.

In order to determine the subcellular compartment in which cleaved granulin peptides are produced, we performed fractionation of fed or starved animals expressing the PGRN-1::RFP reporter. In fed animals, full-length progranulin was enriched in the endolysosomal fraction with very little lower molecular weight granulin observed in any fraction (Fig 3C). Upon starvation, the cleaved granulins increased primarily in the endolysosomal fraction, confirming that the majority of the age and stress-induced granulins are, in fact, endolysosomal (Fig 3C). Therefore, granulin peptides are produced *in vivo* in the endolysosomal compartment in a stress-responsive manner.

Given that granulins impair organismal fitness, localize to the endolysosomal fraction and impair stress resistance, we next investigated their impact on lysosomal morphology. In *C*. *elegans*, coelomocytes scavenge and detoxify the pseudocoelomic cavity and therefore have a well-developed endo-lysosomal system [49]. Although we could not image coelomocyte lysosomes in granulin 1-expressing animals due to the presence of a GFP co-expression marker, we found that both loss of progranulin and expression of granulins 2 and 3 grossly deformed these organelles (Fig 3D–3G). Lysosomes lost their spherical shape, more frequently exhibited membrane protrusions and tubular extensions (Fig 3D–3G), and became smaller in size, reaching significance for *pgrn-1(-)* animals and *pgrn-1(-)*; granulin 3(+) animals (Fig 3H). Together, these data suggest that granulin peptides accumulate in endolysosomes with age and starvation, where they, as well as loss of progranulin, may disrupt lysosomal morphology.

### Granulins impair lysosomal protease activity

**Fig 4:**
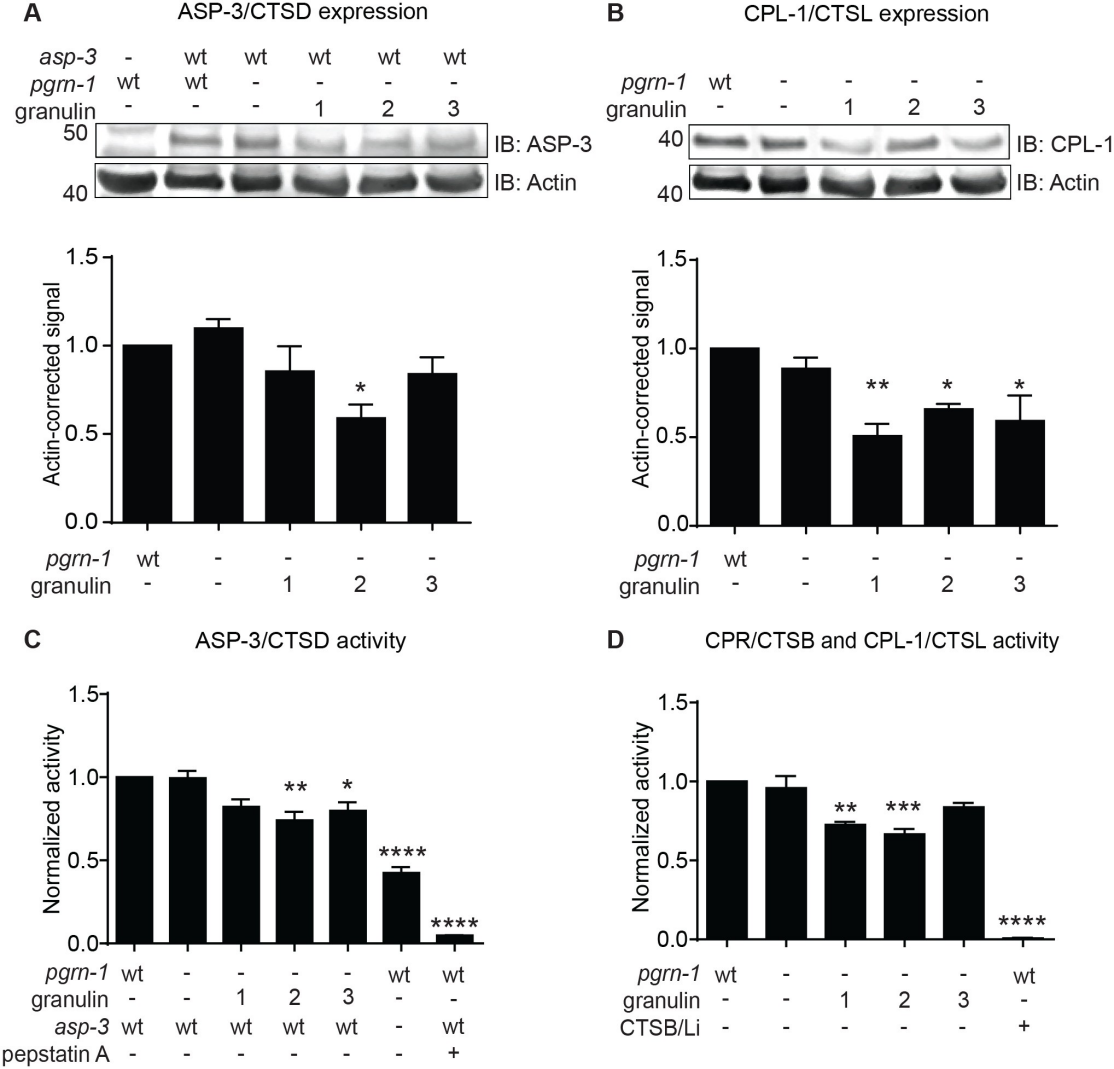
Granulin peptides impair lysosomal protease expression and activity. Total worm lysates from synchronized day 1 adult granulin-expressing animals were immunoblotted with antibodies recognizing (**A**) the aspartyl protease ASP-3/CTSD and (**B**) the cysteine protease CPL-1/CTSL. An anti-actin antibody was used as a loading control. Representative Western blots are shown and data were quantified from 3 independent biological repeats. Enzymatic activity measured in total worm lysates for (**C**) ASP-3/CTSD and (**D**) cysteine protease activity for CPR/CTSB and CPL-1/CTSL. CTSB/Li, cathepsin B and L inhibitor. Data were quantified from 3 independent biological repeats. Throughout, values shown are mean ± SEM, one-way ANOVA with Tukey multiple comparisons test, *P<0.05, **P<0.01, ***P<0.001, ****P<0.0001). Animals lacking *asp-3* continue to have significant protease activity, likely due to other endogenous aspartyl proteases that are inhibited by the pan-aspartyl protease inhibitor, Pepstatin A.

As we observed that expressed granulins disrupt lysosomal morphology, we next assessed their effect on lysosomal function by measuring the expression level and enzymatic activity of lysosomal proteases in lysates from granulin-expressing *C*. *elegans*. Granulin expression resulted in decreased protein levels of ASP-3, the nematode ortholog of mammalian cathepsin D (CTSD), reaching significance in granulin 2-expressing animals (Fig 4A). Expression of all granulins significantly reduced CPL-1 expression, the nematode ortholog of mammalian cathepsin L (CTSL) (Fig 4B). This decrease in protease expression correlated with a decrease in protease activity (Fig 4C and 4D), reaching significance in granulin 2 and 3-expressing animals for ASP-3 activity and granulin 1 and 2-expressing animals for CPR/CPL-1 activity. Overall, our data suggest that granulin peptides disrupt *C*. *elegans* lysosomal protease activity *in vivo*.

### *C*. *elegans* granulin domains display distinct electrostatic and hydrophobicity profiles that may contribute to functional specificity

**Fig 5:**
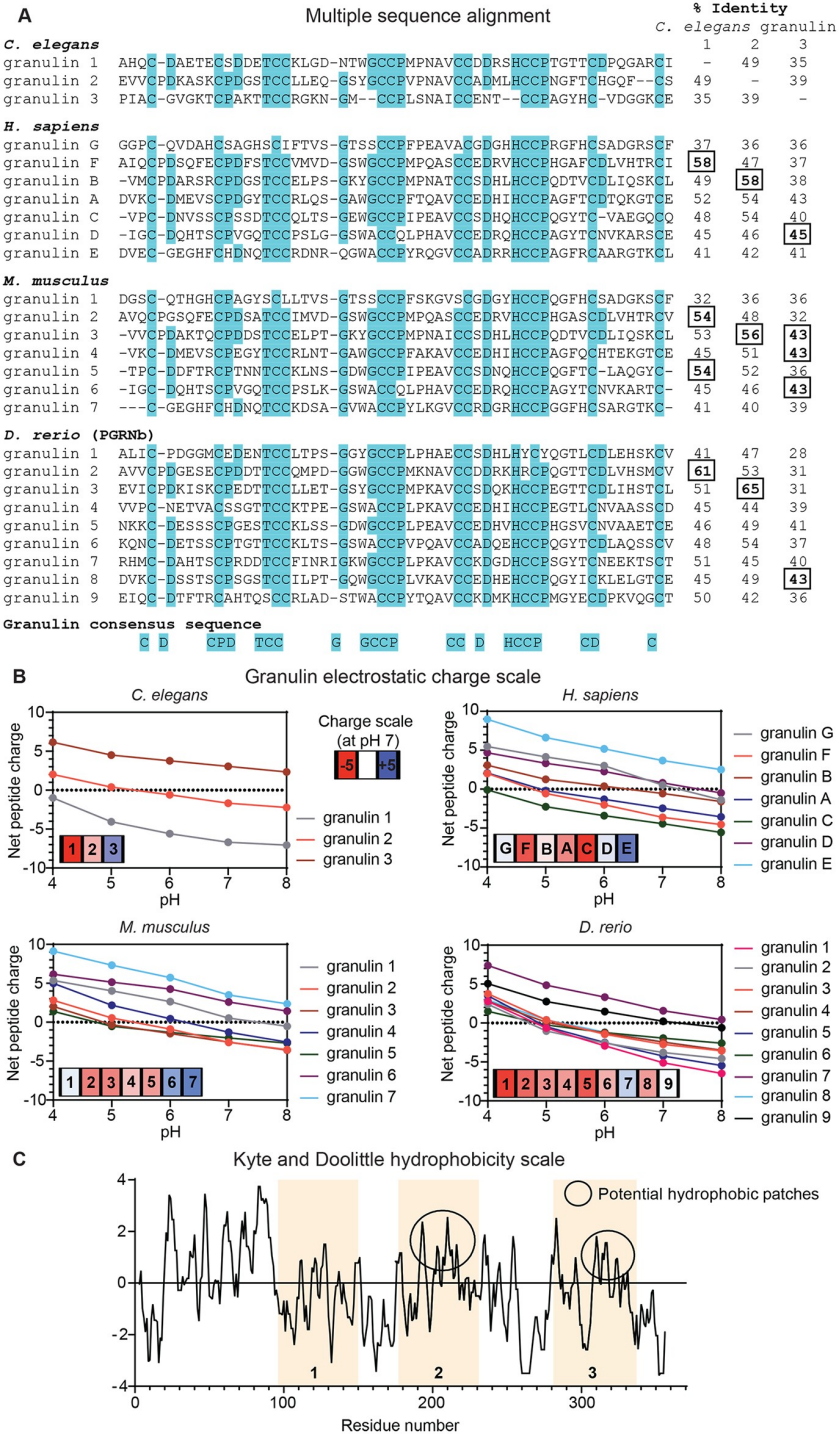
Computational analysis of granulin sequence across species highlights their distinct electrostatic and hydrophobicity profiles. (**A**) Amino acid multiple sequence alignment for *C*. *elegans*, *Homo sapiens*, *Mus musculus and Danio rerio* (PGRNb) granulin domains using the MAFFT (Multiple Alignment using Fast Fourier Transform) server. Residues matching the granulin consensus sequence are highlighted in cyan. The identity score (%) between all granulin domains and *C*. *elegans* granulins 1, 2 and 3 from pairwise sequence alignment using the EMBOSS Needle server is indicated on the right. The granulin domains of different species sharing the highest identity score to *C*. *elegans* granulins 1, 2 and 3 are highlighted in square boxes. (**B**) pH-dependent electrostatic charge scale calculated by propKa 3.1. A 3-color scale is used for the granulin domains, colored from red (negative) to blue (positive) using a percentile scale. (**C**) Kyte and Doolittle (K&D) hydrophobicity scores for granulin domains of *C*. *elegans*. The central regions of granulins 2 and 3 are highlighted with circles since most of these residues have positive K&D scores, which is suggestive of a potential hydrophobic patch.

As we observed differences between the three granulins in terms of the magnitude of their phenotypic effects within assays, we sought to determine whether these differences might be explained by variations in their amino acid sequence and physicochemical properties. *C*. *elegans* granulins 1, 2 and 3 share less than 50% sequence identity among themselves (Fig 5A), and less than 40% when excluding the highly conserved network of disulfide bonds. Electrostatic analysis (Fig 5B) shows that granulin 3, located at the C-terminus of *C*. *elegans* PGRN-1, is positively charged at neutral pH, while granulin 1, the N-terminal granulin domain, remains negatively charged at all analyzed pH values (pH = 4 to 8). The central granulin 2 domain has little to no overall net peptide charge at neutral pH. A further comparison of granulin hydrophobicity (Fig 5C) shows that the central region of granulin 2 (residues 202 to 221) and granulin 3 (residues 309 to 326) is predominantly hydrophobic, as measured by Kyte and Doolittle (K&D) hydrophobicity scores greater than zero. In contrast, the K&D score for the corresponding region of granulin 1 (res. 120 to 139) is slightly negative. While the functions of the individual *C*. *elegans* granulin domains remain to be further elucidated, these observed differences might suggest that each domain participates in unique protein-protein interactions (PPIs), and thus differing roles in the endolysosomal system.

We further compared the *C*. *elegans* granulin sequences with those of different species, including *Homo sapiens (H*. *sapiens)*, *Mus musculus (M*. *musculus)* and *Danio rerio* (*D*. *rerio)* (Fig 5A). We found that *C*. *elegans* granulins share higher identity scores to certain granulins from other species than among themselves. Similar to *C*. *elegans* granulins, differences in pH-dependent electrostatics (Fig 5B) were noticeable for all species studied, with a recurring trend for the C-terminal granulin domains being the most positively charged. The low sequence identity and distinct physicochemical properties among the granulin domains were also observed for *H*. *sapiens*, *M*. *musculus* and *D*. *rerio*, contrasting with the highly conserved network of disulfide bonds. Taken together, these data highlight the importance of the amino acid residues situated outside of the well-conserved granulin sequence consensus for contributing to the charge and hydrophobicity profiles of each granulin domain. These may drive unique recognition patterns for PPIs that may ultimately be relevant in a disease context.

### Granulins activate the lysosomal CLEAR response and induce nuclear localization of HLH-30/TFEB

**Fig 6:**
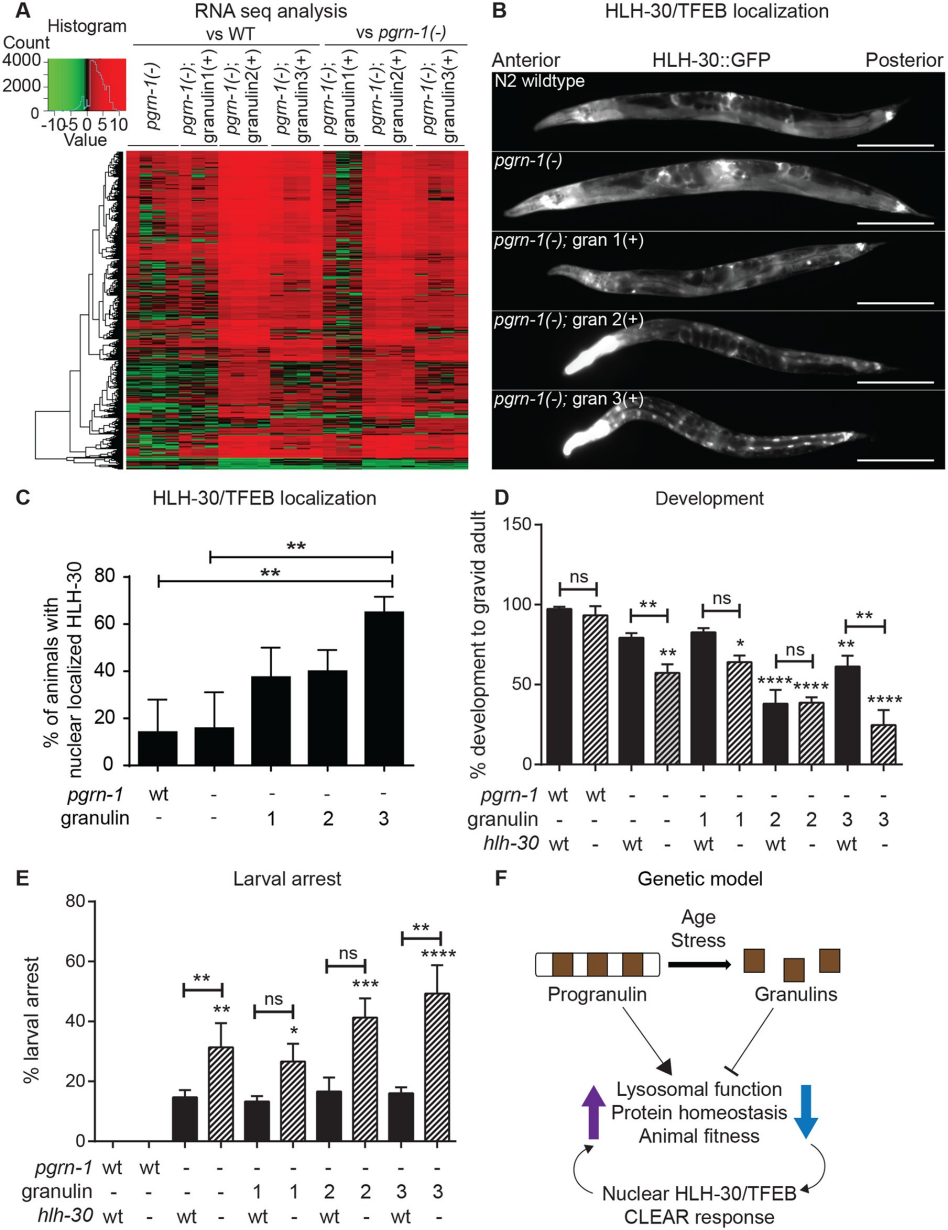
Granulins activate the lysosomal CLEAR response and induce nuclear localization of HLH-30/TFEB. (**A**) Heat map showing the fold-changes of gene expression in comparisons of day 1 adult animals as indicated. Data from four independent biological replicates are shown (except for granulin 1 where one sample was excluded as a quality control outlier). Significance cut-off was a false discovery rate (FDR) of *P*<0.05 (up-regulated = red, down-regulated = green). Number of HLH-30/TFEB binding sites identified/total number of DEGs: *pgrn-1(-)* vs wt: 67/233, *pgrn-1(-)*; granulin 1(+) vs wt: 18/179, *pgrn-1(-)*; granulin 2(+) vs wt: 408/4050, *pgrn-1(-)*; granulin 3(+) vs wt: 1136/2560. See S1–S5 Tables for the complete gene lists. (**B**) Representative images of wild-type, *pgrn-1(-)* and *pgrn-1(-); granulin 3(+)* animals expressing HLH-30::GFP (scale bar = 200 μm). (**C**) Percentage of animals with nuclear localized HLH-30::GFP (n = 120 animals from 3 biological replicates). Wild-type, *pgrn-1(-)* and granulin-expressing animals with and without *hlh-30* expression were staged as embryos, and animals were scored for (**D**) development to gravid adult (n = 50, 3 biological replicates), and (**E**) the number of larvae arresting at L1 and L2 stage (n = 50, 3 biological replicates). Throughout, values shown are mean ± SEM, one-way ANOVA and Tukey multiple comparisons test. Comparisons are to wild-type unless otherwise indicated (*P<0.05, **P<0.01, ***P<0.001, ****P<0.0001, ns = not significant). The ~20% larval arrest observed in the *pgrn-1(-)* strains with wild-type *hlh-30* reach significance when compared pairwise with wildtype using a Student’s t-test (*P<0.05). (**F**) Genetic model for progranulin and granulin function in lysosomal function, protein homeostasis and stress resistance.

To determine if granulin-induced disruption of lysosomal morphology and function promoted a transcriptional response, we performed RNA-seq profiling of wild-type, *pgrn-1(-)* and granulin-expressing animals (S1–S5 Tables). Visual inspection of the RNA sequencing reads confirmed a high and comparable expression of granulin 1, 2 and 3 transgenes, as well as a read drop-out in progranulin null animals (S4A Fig). Wild-type animals had a low but detectable expression of endogenous progranulin transcript (S4A Fig). We first compared *pgrn-1(-)* or *pgrn-1(-)*; granulin animals to wild-type animals. Compared to wildtype, a total of 7084 differentially expressed genes (DEGs) were identified across all strains (Fig 6A and S4B Fig). The majority of DEGs identified for *pgrn-1(-)* animals were down-regulated compared to wild-type animals. These DEGs were enriched for GO terms associated with growth, development, cation and sugar binding (S4C and S4D Fig). In contrast, the majority of DEGs for granulin-expressing animals were up-regulated compared to both wild-type and *pgrn-1(-)* animals (Fig 6A and S4B Fig). GO term analysis for DEGs in granulin-expressing animals showed a shared enrichment in genes associated with lysosomal function, including protein metabolic process and hydrolase activity acting on ester bonds (S4E–S4K and S5 Figs). Expression of granulin 2 resulted in the highest number of DEGs compared to both wild-type and *pgrn-1(-)* animals, followed by granulin 3 and then granulin 1 (Fig 6A and S4B Fig). The observed overlap in enriched GO terms on granulin 2 and 3 expression further suggests similarities between these two granulins compared to granulin 1, and also reflects the phenotype severity observed in development and behavioral assays. Interestingly, the upregulated DEGs identified in *pgrn-1(-)*; granulin 3(+) animals were significantly enriched for genes whose promoters contained the putative TFEB binding site E-box sequence 5’-CACGTG-3’ (*P* = 0.011). This trend was also observed in the upregulated DEGs for *pgrn-1(-)*; granulin 1(+) (*P* = 0.149) and *pgrn-1(-)*; granulin 2(+) (*P* = 0.097) but did not reach statistical significance. TFEB is the master lysosomal transcription factor that regulates lysosomal biogenesis and autophagy [6, 7], and the *C*. *elegans* TFEB is HLH-30 [50].

In response to starvation, stressful stimuli and aging, HLH-30/TFEB translocates from the cytosol to the nucleus to activate its transcriptional targets [6, 7, 50, 51]. This program, known as the Coordinated Lysosomal Expression and Regulation (CLEAR) response induces expression of genes involved in lysosomal function and autophagy, including progranulin. We assessed HLH-30/TFEB cytoplasmic versus nuclear localization in control, *pgrn-1(-)* and granulin expressing animals. Granulin expression promoted nuclear localization of HLH-30/TFEB, reaching significance in granulin 3-expressing animals (Fig 6B and 6C). This effect was not seen in *pgrn-1(-)* animals where a much lower number of DEGs were identified, and was also not observed in *pgrn-1(-)* animals expressing human tau or TDP-43 protein (S6A Fig). These results suggest that the disruption of lysosomal morphology and protein homeostasis seen in granulin-expressing animals leads to a specific compensatory translocation of HLH-30/TFEB from the cytosol to the nucleus.

When granulin-expressing animals were crossed into a wildtype background, the presence of wildtype progranulin partially mitigated the negative effects of granulin-expression on development (S6B Fig), lysosome morphology (S6C and S6D Fig) and HLH-30/TFEB localization (S6E Fig). Interestingly, granulin-expression in a wildtype background resulted in higher ER stress sensitivity than granulin-expression in a progranulin null background (S6F Fig). We speculate that ER stress may promote the cleavage of endogenous PGRN, resulting in even higher levels of cleaved granulins (endogenous *and* transgenic granulins) and enhanced ER stress sensitivity. These data further suggest a reciprocal relationship between full-length progranulin and cleaved granulins, and highlights that their relative levels may be important for normal animal development and fitness.

To determine if the upregulation of TFEB target genes was a compensatory transcriptional response in granulin-expressing animals, we crossed these animals into an *hlh-30(-)* null background. When lacking *hlh-30*, granulin-expressing animals had further impairments in overall fitness, with fewer growing to adulthood (Fig 6D) and more arresting at early larval stages (Fig 6E). Together, these data demonstrate that granulin expression, even in the absence of stress or starvation, is sufficient to activate a compensatory CLEAR response and induce expression of genes containing TFEB binding sites. Overall, the ability of granulins to 1) impair a proteotoxic stress response, 2) disrupt lysosomal morphology, 3) direct TFEB to the nucleus and 4) induce a CLEAR response indicates that granulin-dependent impairment of lysosomal function negatively impacts cellular protein homeostasis (Fig 6F).

## Discussion

We have previously shown in *C*. *elegans* that expression of granulin peptides enhances TDP-43 toxicity and prevents its degradation [39]. In this study, we sought to understand the mechanism by which granulins exert their effects and determine if they more broadly impacted protein homeostasis. We found that granulins are produced in an age and stress-dependent manner, and consequently impair lysosomal protease expression and activity. Their expression negatively impacts cellular protein homeostasis and drives a compensatory lysosomal stress response in an attempt to up-regulate HLH-30/TFEB-regulated genes. These effects manifest as an overall decrease in animal fitness.

This study contributes a new dimension to our understanding of the regulation of lysosomal proteostasis via the identification of *C*. *elegans* granulins as age and stress-produced peptides that impair overall animal fitness by reducing lysosomal function. *C*. *elegans* granulins, similar to the human peptides, localize to the endolysosomal compartment [32]. Granulins are composed of evolutionarily conserved stacked beta hairpins stabilized by disulfide bonds, which are often found in natural protease inhibitors [52]. This highly compact and stable structure is thought to confer resistance to denaturation and protection against proteolytic cleavage in the lysosomal environment [53]. Indeed, a role for granulins in regulating protease maturation has previously been demonstrated in plant cysteine proteases that incorporate a granulin domain C-terminal to the catalytic domain, such as RD21 in *A*. *thaliana* [54]. In further support of granulins as regulators of protease activity, homozygous progranulin mutation carriers develop a progressive myoclonic epilepsy syndrome that phenocopies loss of function mutations in another lysosomal protease inhibitor, cystatin B [18, 55]. Recent studies have shown that human full-length progranulin and individual granulin domains may physically interact with CTSD and stimulate the enzymatic activity of the protease [25, 56–58]. However, in the absence of full-length protein, *C*. *elegans* granulins promote a distinct phenotype of impaired resistance to ER stress, delayed growth, decreased CTSD and CTSB/L activity and activation of the CLEAR transcriptional program.

Granulins likely play a *normal physiological role* in regulating protease expression and activity. Given their ability to promote the CLEAR program, granulins may serve as a signal for stress or impaired health that requires regulated checks on protease activity, perhaps to limit inflammation. This would be consistent with the role of progranulin in complement-mediated synaptic pruning by microglia [59]. We speculate that under conditions of progranulin haploinsufficiency, the normal balance between progranulin and granulins becomes skewed towards excessive granulins. In excess, the inhibitory effect of granulins upon protease activity impairs the function of lysosomes; with age, the natural compensatory mechanisms such as the CLEAR program become overwhelmed, resulting in cellular dysfunction. When this occurs in neurons and/or support cells such as microglia, the end result may be neurodegeneration. Because granulins increase with age, it remains possible that accumulation of granulins directly contribute to the proteostatic pressures associated with increasing age. Comprehensive measures of progranulin-to-granulin ratios with age and in progranulin mutation carriers are needed.

The lentiviral delivery of progranulin to degenerating brain regions protects against neurotoxicity and cognitive defects in mouse models of Parkinson’s disease [60] and Alzheimer’s disease [61]. As such, efforts to increase progranulin production in patients are underway [62–65]. However, a more recent study has suggested that progranulin delivery to brain promotes in T-cell infiltration and neuronal and glial degeneration [66]. Progranulin cleavage and granulin levels were not measured in these studies, and may account for differences in the observed results.

Progranulin is a highly conserved protein [27, 29, 30]. The number of granulin domains has increased through phylogeny from one in *Dictyostelium discoideum* and plants, three in nematodes to seven-and-a-half in humans [29, 54]. It is intriguing to speculate that this expansion in cleavage fragments could lead to regulation of additional proteases. In support of this, we find that the amino acid residues situated outside of the well-conserved granulin sequence consensus contribute to distinct charge and hydrophobic profiles for each granulin domain. These unique characteristics may be important for driving specific protein-protein interactions and thus different roles in the cellular environment. Indeed, the distinct effects of granulin 2 and 3 on protein homeostasis, lysosomal function and TDP-43 toxicity [39], as compared to granulin 1, may suggest functional differences between granulins.

Our results establish age-regulated granulins as modulators of lysosomal function, and suggest that a *toxic gain of granulin function*, rather than or in addition to simply loss of full-length progranulin, may contribute to FTLD disease pathogenesis. This could explain why progranulin loss-of-function mutations are transmitted in an autosomal dominant fashion. The presence of granulins only in the haploinsufficiency state could explain why TDP-43 pathology is not seen in the null state [18]. Several lysosomal proteases that cleave progranulin have recently been identified [32–34], although how those proteases decide when and where to cleave progranulin remains unknown. This study prompts several important follow up questions regarding the rate and order in which granulins are liberated from progranulin, how pH changes impact the predicted association of granulins with lysosomal proteases and whether increased granulin impact other neurodegenerative disorders such as Alzheimer’s disease. The current study also has implications for therapeutic progranulin repletion efforts, as care should be taken to determine whether replacement progranulin is processed into granulins. Finally, our findings suggest that in addition to progranulin repletion, prevention of progranulin cleavage into granulins could represent a rational therapeutic target in neurodegeneration.

## Materials and methods

### Strains

*C*. *elegans* strains were cultured at 20 °C according to standard procedures [67]. Some strains were provided by the Mitani Laboratory (National Bioresource Project, Japan) at the Tokyo Women’s Medical University and the Caenorhabditis Genetics Center (CGC) at the University of Minnesota. Strain descriptions are at www.wormbase.org. The N2E control strain was used as the wild-type strain. The *pgrn-1(tm985)* strain has a 347 bp deletion in the *pgrn-1* gene resulting in a null allele [45]. The following *C*. *elegans* strains were used in this study:

CF3050 *pgrn-1(tm985) I*

AWK33 *pgrn-1(tm985) I; rocIs1[Ppgrn-1+SignalSequence*::*granulin1*::*FLAG*::*polycistronic mCherry + Punc-122*::*GFP]*

AWK43 *pgrn-1(tm985) I*; *rocEx14[Ppgrn-1+SignalSequence*::*granulin2*::*FLAG*::*polycistronic mCherry + Pmyo-2*::*GFP]*

AWK107 *pgrn-1(tm985) I*; *rocIs5[Ppgrn-1+SignalSequence*::*granulin3*::*FLAG*::*polycistronic mCherry + Pmyo-2*::*GFP]*

AWK308 N2E*; rocIs1[Ppgrn-1+SignalSequence*::*granulin1*::*FLAG*::*polycistronic mCherry + Punc-122*::*GFP]*

AWK309 N2E; *rocEx14[Ppgrn-1+SignalSequence*::*granulin2*::*FLAG*::*polycistronic mCherry + Pmyo-2*::*GFP]*

AWK310 N2E; *rocIs5[Ppgrn-1+SignalSequence*::*granulin3*::*FLAG*::*polycistronic mCherry + Pmyo-2*::*GFP]*

AWK459 *pgrn-1(tm985) I; muIs216[Paex-3*::*huMAPT 4R1N +Pmyo-3*::*RFP]*

CF3588 *pgrn-1(tm985) I; muIs206[Pegl-3*::*TDP-43*::*GFP]*

AWK524 *pgrn-1(tm985) I; muIs189[Ppgrn-1*::*pgrn-1*::*polycishronic mCherry +Podr-1*::*CFP]*

AWK466 *pgrn-1(tm985) I; muIs189[Ppgrn-1*::*pgrn-1*::*polycishronic mCherry +Podr-1*::*CFP]*; *rocEx14[Ppgrn-1+SignalSequence*::*granulin2*::*FLAG*::*polycistronic mCherry + Pmyo-2*::*GFP]*

CF3778 *pgrn-1(tm985) I; muIs213[Ppgrn-1*::*pgrn-1*::*RFP]*

AWK181 *pgrn-1(tm985) I; unc-119(ed3)III; pwIs503[vha6p*::*mans*::*GFP + Cb unc-119(+)]; muIs213[Ppgrn-1*::*pgrn-1*::*RFP]*

AWK360 *pgrn-1 (tm985) I; unc-119(ed3) III; pwIs50[Plmp-1*::*lmp-1*::*GFP + Cbr-unc-119(+)]; muIs213[Ppgrn-1*::*pgrn-1*::*RFP]*

AWK395 *pgrn-1 (tm985) I; unc-119(ed3) III; cdIs54[pcc1*::*MANS*::*GFP + unc-119(+) + myo-2*::*GFP]; muIs213[Ppgrn-1*::*pgrn-1*::*RFP]*

AWK374 *pgrn-1 (tm985) I; bIs34[rme-8*::*GFP + rol-6(su1006)]; muIs213[Ppgrn-1*::*pgrn-1*::*RFP]*

MAH235 *sqIs19[Phlh-30*::*hlh-30*::*gfp + rol-6(su1006)]*

AWK403 *pgrn-1(tm985) I; sqIs19[Phlh-30*::*hlh-30*::*gfp + rol-6(su1006)]*

AWK404 *pgrn-1(tm985) I; sqIs19[Phlh-30*::*hlh-30*::*gfp + rol-6(su1006)]; rocIs1[Ppgrn-1+SS*::*granulin1*::*FLAG*::*polycistronic mCherry]*

AWK405 *pgrn-1(tm985) I; sqIs19[Phlh-30*::*hlh-30*::*gfp + rol-6(su1006)]; rocEx14[Ppgrn-1+SS*::*granulin2*::*FLAG*::*polycistronic mCherry + Pmyo-2*::*GFP]*

AWK406 *pgrn-1(tm985) I; sqIs19[Phlh-30*::*hlh-30*::*gfp + rol-6(su1006)]; rocIs5[Ppgrn-1+SS*::*granulin3*::*FLAG*::*polycistronic mCherry + Pmyo-2*::*GFP]*

AWK467 N2E*; sqIs19[Phlh-30*::*hlh-30*::*gfp + rol-6(su1006)]; rocIs1[Ppgrn-1+SS*::*granulin1*::*FLAG*::*polycistronic mCherry]*

AWK469 N2E*; sqIs19[Phlh-30*::*hlh-30*::*gfp + rol-6(su1006)]; rocEx14[Ppgrn-1+SS*::*granulin2*::*FLAG*::*polycistronic mCherry + Pmyo-2*::*GFP]*

AWK471 N2E*; sqIs19[Phlh-30*::*hlh-30*::*gfp + rol-6(su1006)]; rocIs5[Ppgrn-1+SS*::*granulin3*::*FLAG*::*polycistronic mCherry + Pmyo-2*::*GFP]*

AWK546 *pgrn-1(tm985) I; sqIs19[Phlh-30*::*hlh-30*::*gfp + rol-6(su1006)]; muIs216[Paex-3*::*huMAPT 4R1N +Pmyo-3*::*RFP]*

AWK547 *pgrn-1(tm985) I; sqIs19[Phlh-30*::*hlh-30*::*gfp + rol-6(su1006)]; muIs206[Pegl-3*::*TDP-43*::*GFP]*

JIN1375 *hlh-30(tm1978) IV*

AWK514 *pgrn-1 (tm985) I; hlh-30(tm1978) IV*

AWK516 *pgrn-1 (tm985) I; hlh-30(tm1978) IV; rocIs1[Ppgrn-1+SS*::*granulin1*::*FLAG*::*polycis mCherry]*

AWK518 *pgrn-1 (tm985) I; hlh-30(tm1978) IV; rocEx14 [Ppgrn-1+SS*::*granulin2*::*FLAG*::*polycistronic mCherry + Pmyo-2*::*GFP]*

AWK519 *pgrn-1 (tm985) I; hlh-30(tm1978) IV; rocIs5 [Ppgrn-1+SS*::*granulin3*::*FLAG*::*polycistronic mCherry + Pmyo-2*::*GFP]*

AWK521 *pgrn-1 (tm985) I; hlh-30(tm1978) IV; muIs189[Ppgrn-1*::*pgrn-1*::*polycistronic mCherry +Podr-1*::*CFP]*

AWK296 N2E; *Ex[Pced-1*::*asp-3*::*mrfp + pRF4(rol-6)]; unc-119(ed3) III; pwIs50[Plmp-1*::*lmp-1*::*GFP + Cbr-unc-119(+)]*

AWK333 *pgrn-1(tm985) I; Ex[Pced-1*::*asp-3*::*mrfp + pRF4(rol-6)]; unc-119(ed3) III; pwIs50[Plmp-1*::*lmp-1*::*GFP + Cbr-unc-119(+)]*

AWK247 *pgrn-1(tm985) I; pwls50[lmp-1*::*GFP + Cbr-unc-119(+)];rocEx14 [Ppgrn-1+SS*::*granulin2*::*FLAG*::*polycis tronic mCherry + Pmyo-2*::*GFP]*

AWK334 *pgrn-1(tm985) I; Ex[Pced-1*::*asp-3*::*mrfp + pRF4(rol-6)]; unc-119(ed3) III; pwIs50[Plmp-1*::*lmp-1*::*GFP + Cbr-unc-119(+)]; rocIs5[Ppgrn-1+SS*::*granulin3*::*FLAG*::*polycis tronic mCherry + Pmyo-2*::*GFP]*

AWK177 *asp-3(tm4450) X*

VM487 *nmr-1(ak4)II*

### Generation of transgenic *C*. *elegans*

To generate strains expressing individual granulins, each granulin was amplified separately from wild-type *C*. *elegans* progranulin cDNA as previously described [39].

### ER stress assays

ER stress assays were performed as previously described [40].

### Animal viability

L4 stage animals were allowed to lay eggs overnight. Fifty synchronized eggs were transferred to seeded plates. After three days, the fraction of animals that developed to the L4 stage was quantified.

### Body size

L4 animals were staged, grown at 20 °C overnight and imaged the following day as day 1 adults. Animals were mounted on a 2% agarose pad with 25 mM sodium azide (Spectrum Chemical, #SO110) and imaged using a Zeiss AxioImager microscope at 10 x. Body size was measured in ImageJ software using the skeletonize function.

### Short-term associative learning

Short-term associative learning assays were performed as previously described [43, 44].

### Immunoblotting

Sixty L4 stage animals were allowed to lay eggs overnight (~sixteen hours). Adult worms and hatched larvae were washed off the plates with M9 buffer. Eggs were collected with a cell scraper and transferred to a newly seeded plate by chunking. These eggs were allowed to develop to early L4 stage and 200 μl of 20 mM FUDR (Fisher Scientific, #AC227601000) was added to prevent development of progeny and overgrowth of plates. At each time point, animals were collected from plates with ice cold M9 and washed once to remove food. The worm pellet was resuspended 1:1 in freshly made ice cold RIPA buffer (50 mM Tris pH 7.4, 150 mM NaCl, 5 mM EDTA, 0.5% SDS, 0.5% SDO, 1% NP-40, 1 mM PMSF, cOmplete protease inhibitor (Roche, #04693124001) and PhosSTOP phosphatase inhibitor (Roche, #04906837001), 0.3 mM Pefabloc (Roche, #11429868001)). Worms were transferred to Eppendorf tubes and sonicated for 4 cycles of 1 minute on and 2 minutes off (BioRuptor, Diagenode). Lysates were centrifuge for 5 minutes at 13,000 rpm at 4 °C. Supernatant was transferred to a fresh Eppendorf tube and samples were boiled at 95 °C (with 4x LDS, 10% reducing agent) for 5 minutes and analyzed by SDS PAGE. 10–50 μg total protein was resolved on 4–12% gradient SDS-PAGE gels and transferred to PVDF.

### Antibodies

Commercial antibodies used for Western blotting were the following:

Anti-HSP-4/BiP (Novus Biologicals, #NBP1-06274, 1:1000 dilution)

Anti-RFP (GenScript, #A00682, 1:1000 dilution)

Anti-FLAG (Sigma, #F3165, 1:1000 dilution)

Anti-LMP-1(Developmental Studies Hybridoma Bank, #LMP1, 1:100 dilution)

Anti-HSP-70/HSC-70 (Santa Cruz Biotechnology Inc., #sc-33575, 1:1000 dilution)

Anti-calnexin (Novus Biologicals, #NBP1-97476, 1:1000 dilution)

Anti-CPL-1 (Abcam, #ab58991, 1:500 dilution)

Anti-actin (EMD Millipore, #MAB1501R, 1:5000 dilution)

Goat anti-mouse (LI-COR IRDye 800CW, #925–32210, 1:10,000 dilution)

Goat anti-rabbit (LI-COR IRDye 800CW, #925–32211, 1:10,000 dilution)

Donkey anti-goat (LI-COR IRDye 800CW, #925–32214, 1:10,000 dilution)

Donkey anti-mouse (LI-COR IRDye 680RD, #925–68072, 1:10,000 dilution)

Antibodies made in-house and used for Western blotting were the following:

Anti-granulin 1(RB2481, Biomatik, epitope HQCDAETEC(acm)SDDET, 1:1000 dilution)

Anti-granulin 3 (RB2487, Biomatik, epitope CTVLMVESARSTLKL, 1:1000 dilution)

Anti-ASP-3 (Fred Hutchinson, epitope CTGPTDVIKKIQHKIG, 1:1000 dilution)

Imaging and quantification were performed on the LI-COR Odyssey Infrared System. Three independent blots were performed.

### Confocal microscopy

Animals were mounted on microscope slides with 2% agarose pads containing 30 mM levamisole hydrochloride (Fisher Scientific, #AC187870100) and imaged using a Zeiss LSM 700 laser-scanning confocal microscope using 488 nm and 561 nm lasers and 63x and 100x objectives. L1 animals were imaged 1–2 h after hatching. Z-stacks were taken every 0.7 μm. Image processing was carried out using ImageJ software. A maximum intensity projection of the z-stack for each animal was created. Images at 488 nm and 561 nm were overlaid and analyzed for co-localization.

### Subcellular fractionation

Thirty L4 stage animals were picked to 60 x 10 cm plates per strain. Plates were confluent with mixed stage animals after four days growth at 20 °C. Progranulin cleavage was observed after starving animals for an additional seventy-two hours at 20 °C. A lysosomal fraction was isolated from a light mitochondrial-lysosomal fraction as previously described [68] with the following modifications. Animals were collected in 0.25 M sucrose (pH 7.2) and washed twice with 0.25 M sucrose. Lysosomes and mitochondria were separated using a discontinuous Nycodenz (Progen Biotechnik, Germany, #1002424) density gradient. Lysosomes were collected from the 19.8% / sucrose interface and the 26.3 / 19.8% interface and pooled. Lysosomes were diluted five times with 0.25 M sucrose, and pelleted at 37,000 × *g* for 15 minutes. Cytosolic, ER and lysosomal fractions were confirmed by immunoblotting for specific subcellular fraction markers (LAMP-1, HSC-70, calnexin).

### Protease activity measurements from total worm lysates

Protease activity was measured using commercially available kits (BioVision Cathepsin D Activity Fluorometric Assay Kit, #K143-100 and BioVision Cathepsin L Activity Fluorometric Assay Kit, #K142-100). Animals were staged as for immunoblotting, but without the addition of 20 mM FUDR. At day 1 of adulthood, worms were collected from plates with ice cold M9 and washed twice to remove food. Worm pellets were resuspended in 1% NP-40 buffer (Fisher Scientific) without protease inhibitors and frozen at -80 °C overnight. Pellets were thawed and sonicated for 4 cycles of 1 min on and 2 min off (BioRuptor, Diagenode). Lysates were centrifuged for 5 minutes at 13,000 rpm at 4 °C and supernatant was transferred to a fresh tube. 0.25 μg total protein per sample was used per assay and samples from one strain were run in triplicate. Fluorescence measurements were taken every minute at 25 °C (Infinite M200, Tecan). As controls, 250 nM Pepstatin A (for pan-aspartyl protease inhibition in CTSD assay, BioVision) or 10 μM CA-074 (for Cathepsin B inhibition, EMD Millipore, #205530) and 10 μM CTSLiII (for Cathepsin L inhibition, EMD Millipore, #219426) were added to the lysate and pre-incubated for 10 minutes on the bench at room temperature. Linear regression was performed on at least 30 minutes of data to calculate the rate of enzyme activity.

### Computational analysis of granulin domains

Sequences for *C*. *elegans* (Q9U362), *Homo sapiens* (P28799) and *Mus musculus* (P28798) PGRN were extracted from Uniprot (The Uniprot Consortium, 2019), while *Danio rerio* PGRNb (AAH96854.1) sequence was obtained from National Center for Biotechnology Information (NCBI) Protein database (https://www.ncbi.nlm.nih.gov). Amino acid multiple sequence alignment was performed using the MAFFT online service (version 7, https://mafft.cbrc.jp/alignment/server/) [69]. The EMBOSS Needle server was used for pairwise sequence alignment between *C*. *elegans* granulin 1, granulin 2 and granulin 3 and individual granulin domains from *H*. *sapiens*, *M*. *musculus and D*. *rerio (*https://www.ebi.ac.uk/Tools/psa/emboss_needle/) [70]. Identification of granulin domains from the full-length sequences was based on sequence similarity to *H*. *sapiens* granulin A using the Basic Local Alignment Search Tool protein (BLASTp) server (https://blast.ncbi.nlm.nih.gov/Blast.cgi). Granulin A (PDB ID: 2JEY.A) was used as a reference for homology modeling of all granulin domains [71] using the Prime software. Electrostatic analysis ranging from pH 4 to 8 was performed on the *in silico* models with the software propKa 3.1 [72]. Kyte & Doolitle (K&D) hydrophobicity scales were obtained from the ExPASy Bioinformatics Resource Portal (https://web.expasy.org) for PGRN sequence of all species here studied. For the K&D per-residue score, a window size of 5 was used, i.e. the final score for a given residue i is the sum of the scale values for i and i-2, i-1, i+1 and i+2.

### RNA-sequencing analysis

Total RNA was isolated from wild-type (N2E), *pgrn-1(-)*, *pgrn-1(-)*; granulin 1(+), *pgrn-1(-)*; granulin 2(+) and *pgrn-1(-)*; granulin 3(+) expressing animals synchronized at day 1 of adulthood. Animals were collected from plates with ice cold M9 and washed three times to remove OP50 food. After harvesting, the animals were resuspended in QIAzol (Qiagen #79306) and flash frozen in liquid nitrogen. RNA was extracted and purified using a Qiagen miRNeasy kit (Qiagen #217004). Samples were extracted in quadruplicate (four biological replicates for each strain), for a total of 20 samples. Total RNA was quantified using the RiboGreen assay (ThermoFisher, #R11490) and RNA quality was checked using an Agilent TapeStation 4200 (Agilent). RNA Integrity Numbers (eRINs) were >8 in all the samples. Libraries for RNA-seq were prepared using the Illumina TruSeq library preparation protocol (Illumina Inc), multiplexed into a single pool and sequenced using an Illumina HiSeq 4000 sequencer across 4 PE 2 x 75 lanes on a single flowcell. After demultiplexing, we obtained between 13 and 32 million reads per sample, each one 75 paired end bases long. Quality control was performed on base qualities and nucleotide composition of sequences. Alignment to the *C*. *elegans* genome (ce11) was performed using the STAR spliced read aligner [73] with default parameters. Additional QC was performed after the alignment to examine the following: level of mismatch rate, mapping rate to the whole genome, repeats, chromosomes, and key transcriptomic regions (exons, introns, UTRs, genes). Between 92 and 93% of the reads mapped uniquely to the worm genome. Total counts of read fragments aligned to candidate gene regions within the *C*. *elegans* reference gene annotation were derived using HTS-seq program and used as a basis for the quantification of gene expression. Only uniquely mapped reads were used for subsequent analyses. Following alignment and read quantification, we performed quality control using a variety of indices, including sample clustering, consistency of replicates, and average gene coverage. One sample for *pgrn-1(-)*; *granulin 1(+)* was excluded from analysis as a quality control outlier. Differential expression analysis was performed using two parallel approaches, the EdgeR Bioconductor package [74], and voom [75]. Differentially expressed genes (DEGs) were selected based on False Discovery Rate (FDR, Benjamini-Hochberg adjusted p-values) estimated at ≤ 5%. There was a large overlap between DEGs identified by edgeR and voom (edgeR: 89.0% common DEGs with voom (6307/7084), voom: 93.9% common DEGs with edgeR (6307/6714)). Clustering and overlap analyses were performed using the Bioconductor packages within the statistical environment R (www.rproject.org/). Gene Ontology annotation was performed using DAVID (david.abcc.ncifcrf.gov/) and GOrilla [76, 77].

### TFEB binding site analysis

The promoter regions of all differentially regulated transcripts were analyzed for the presence of the *C*. *elegans* TFEB/HLH-30 binding site E-box sequence 5’-CACGTG-3’. Enrichment of TFEB binding sites was tested by comparison to the expected distribution based on 10,000 random permutations. A permutation test was used to calculate p-values.

### HLH-30/TFEB imaging

Forty L4 animals were picked, grown at 20 °C overnight and imaged the following day as day 1 adults. The nuclear localization of HLH-30::GFP was imaged using a Zeiss AxioImager microscope at 10x. Animals were imaged within 5 minutes of mounting on a 2% agarose pad with 25mM sodium azide (Spectrum Chemical, #SO110). Data from three independent experiments were pooled.

## Supporting information

S1 Fig *C*. *elegans* granulins specifically impair resistance to ER stress. (**A**) Total worm lysates from synchronized day 1 adult animals over-expressing *C*. *elegans* progranulin (CePGRN-1 OE) were immunoblotted with an anti-granulin 1 antibody after being grown in the presence and absence of 5μg tunicamycin (3 biological replicates, TM = tunicamycin). Anti-HSP-4/BiP, the nematode homolog of human BiP/Grp78, was used to confirm the induction of ER stress and anti-actin was used as a loading control. (**B**) Quantification of HSP-4/BiP expression in the presence and absence of 5μg tunicamycin in *pgrn-1(-)* animals (3 biological replicates, error bars show mean ± SEM). (**C**) Quantification of HSP-4/BiP expression in the presence and absence of 5μg tunicamycin in animals with *C*. *elegans* progranulin over-expression (3 biological replicates, error bars show mean ± SEM). (**D**) Quantification of CePGRN-1 expression in the presence and absence of 5μg tunicamycin in animals with *C*. *elegans* progranulin OE (3 biological replicates, error bars show mean ± SEM). (**E**) Wild-type (N2) and *pgrn-1(-)* animals with and without *C*. *elegans* granulin 2 and progranulin over-expression were subjected to ER stress with tunicamycin (2 μg / ml). The fraction developing to L4 stage was quantified (n = 50, 3 biological replicates). Granulin 1 and 3 could not be tested because these transgenes are on the same chromosome as the progranulin over-expression transgene and recombinants were not obtained from the crosses. (**F**) Wild-type (N2) and *pgrn-1(-)* animals with and without *C*. *elegans* granulin 3, human 1N4R tau and human TDP-43 over-expression were subjected to ER stress with tunicamycin (5 μg / ml). The fraction developing to L4 stage was quantified (n = 50, 3 biological replicates).
(TIF)

S2 Fig Expressed granulins are found within lysosomes. (**A-C**) Validation of subcellular fractions from *C*. *elegans* by blotting for fraction-specific markers: (**A**) anti-LMP-1 is specific for the lysosomal fraction, (**B**) anti-HSP-70 which is normally found in both cytosolic and lysosomal fractions, and (**C**) calnexin which localizes to the ER fraction. Similar results were observed in three independent Western blots. WL = whole lysate, C = cytosol, L = lysosomes, ER = endoplasmic reticulum.
(TIF)

S3 Fig Progranulin expression and cleavage is stress-responsive. (**A-D**) Quantification of actin-corrected mean band intensities from Western blot of *C*. *elegans* PGRN-1::RFP lysates with (**A-B**) aging, and (**C-D**) starvation. Actin-corrected mean band intensities were normalized to highest value per experiment (data from three biological replicates is shown, values shown are mean ± SEM, one-way ANOVA and Tukey multiple comparisons test, **P<0.01, ***P<0.001, ****P<0.0001). (**E**) In *C*. *elegans pgrn-1(-)*; PGRN-1::RFP lysates the most prominent cleavage product at ~30 kDa was recognized by both granulin 3 and RFP antibodies. (**F**) Subcellular fractionation of *pgrn-1(-)*; PGRN::RFP animals. Whole lysate (WL), cytosol (C), lysosome (L) and endoplasmic reticulum (ER) fractions from starved (70 hours off-food) animals were immunoblotted with anti-RFP and anti-LMP-1 antibodies.
(TIF)

S4 Fig GO enrichment analysis highlights shared terms between granulin-expression compared to wildtype animals. (**A**) RNA sequencing read counts at the progranulin gene locus (n = 4). Error bars show the mean ± SEM, one-way ANOVA with post-hoc Tukey multiple comparisons test. (**B**) Numbers of differentially expressed genes (DEGs) identified in the comparisons indicated. (**C-D**) GO term enrichment analysis for *pgrn-1(-)* animals compared to wildtype (WT) for (**C**) Biological Process (BP) and (**D**) Molecular Function (MF) categories. (**E**) GO term enrichment analysis for *pgrn-1(-)*; granulin 1(+) animals compared to WT for BP. (**F-H**) GO term enrichment analysis for *pgrn-1(-)*; granulin 2(+) animals compared to WT for (**F**) BP, (**G**) MF and (**H**) Cellular Component (CC) categories. (**I-K**) GO term enrichment analysis for *pgrn-1(-)*; granulin 3(+) animals compared to WT for (**I**) BP, (**J**) MF and (**K**) CC categories. For all panels, data from four independent biological replicates are shown (except for granulin 1 where one sample was excluded as a quality control outlier). The significance cut-off was a false discovery rate (FDR) of *P*<0.05, up-regulated = red, down-regulated = green. For C-K, the number of DEGs identified within each GO term is indicated in parentheses.
(TIF)

S5 Fig GO enrichment analysis highlights shared terms between granulin 2 and 3 expression compared to progranulin null animals. (**A-C**) GO term enrichment analysis for *pgrn-1(-)*; granulin 2(+) animals compared to *pgrn-1(-)* for (**A**) Biological Process (BP), (**B**) Molecular Function (MF) and (**C**) Cellular Component (CC) categories. (**D-F**) GO term enrichment analysis for *pgrn-1(-)*; granulin 3(+) animals compared to WT for (**D**) BP and (**E**) MF and (**F**) CC categories. For all panels, data from four independent biological replicates are shown. The significance cut-off was a false discovery rate (FDR) of *P*<0.05, up-regulated = red, down-regulated = green. The number of DEGs identified within each GO term is indicated in parentheses.
(TIF)

S6 Fig Granulin-induced impairment of development, lysosome morphology and HLH-30/TFEB localization is diminished in the presence of wildtype progranulin. (**A**) Percentage of animals with nuclear localized HLH-30::GFP (n = 90 animals from 3 biological replicates). Representative images of *pgrn-1(-); tau(+)* and *pgrn-1(-); TDP-43(+)* animals expressing HLH-30::GFP are shown (scale bar = 200 μm). (**B**) Wild-type (N2) and *pgrn-1(-)* animals with and without granulin expression were staged as embryos. Animals were scored for development to L4 stage (n = 50, 3 biological replicates). (**C**) Representative light and fluorescent confocal images of anterior coelomocyte cells expressing LMP-1::GFP in N2; granulin 2(+) and N2; granulin 3(+) animals. Scale bars are 10 μm. Dashed white lines mark the outline of each coelomocyte cell. (**D**) Lysosomal diameter measurements from anterior coelomocyte cells (n = 60). Mean values (μm): wt: 1.57 ± 0.04, *pgrn-1(-)*: 1.37 ± 0.05, N2; granulin 2(+): 1.62 ± 0.04, *pgrn-1(-)*; granulin2(+): 1.47 ± 0.05, N2; granulin 3(+): 1.23 ± 0.03, *pgrn-1(-)*; granulin3(+): 1.16 ± 0.05. (**E**) Percentage of animals with nuclear localized HLH-30::GFP (n = 120 animals from 4 biological replicates). (**F**) Wild-type (N2) and *pgrn-1(-)* animals with and without granulin expression were subjected to ER stress with tunicamycin (5 μg / ml). The fraction developing to L4 stage was quantified (n = 50, 3 biological replicates). For panels A, B, C, E and F, error bars show the mean ± SEM, one-way ANOVA with post-hoc Tukey multiple comparisons test. Comparisons are to wildtype unless otherwise indicated (*P<0.05, **P<0.01, ***P<0.001, ****P<0.0001, wt = wildtype, ns = not significant).
(TIF)

S1 Table Identification of enriched GO terms on progranulin loss and granulin expression through edgeR and DAVID analysis. (See corresponding Excel file).
(XLSX)

S2 Table Identification of enriched GO terms on progranulin loss and granulin expression through edgeR and GOrilla analysis. (See corresponding Excel file).
(XLSX)

S3 Table Identification of enriched GO terms on progranulin loss and granulin expression through voom and DAVID analysis. (See corresponding Excel file).
(XLSX)

S4 Table Identification of enriched GO terms on progranulin loss and granulin expression through voom and GOrilla analysis. (See corresponding Excel file).
(XLSX)

S5 Table Identification of TFEB binding sites in differentially regulated transcripts from progranulin loss and granulin expression. (See corresponding Excel file).
(XLSX)

## References

[1] David DC, Ollikainen N, Trinidad JC, Cary MP, Burlingame AL, Kenyon C. Widespread protein aggregation as an inherent part of aging in C. elegans. PLoS biology. 2010;8(8):e1000450 10.1371/journal.pbio.1000450 20711477 PMC2919420

[2] Reis-Rodrigues P, Czerwieniec G, Peters TW, Evani US, Alavez S, Gaman EA, et al Proteomic analysis of age-dependent changes in protein solubility identifies genes that modulate lifespan. Aging cell. 2012;11(1):120–7. 10.1111/j.1474-9726.2011.00765.x 22103665 PMC3437485

[3] Walther DM, Kasturi P, Zheng M, Pinkert S, Vecchi G, Ciryam P, et al Widespread Proteome Remodeling and Aggregation in Aging C. elegans. Cell. 2015;161(4):919–32. 10.1016/j.cell.2015.03.032 25957690 PMC4643853

[4] Jackson MP, Hewitt EW. Cellular proteostasis: degradation of misfolded proteins by lysosomes. Essays in biochemistry. 2016;60(2):173–80. 10.1042/EBC20160005 27744333 PMC5065703

[5] Stoka V, Turk V, Turk B. Lysosomal cathepsins and their regulation in aging and neurodegeneration. Ageing research reviews. 2016;32:22–37. 10.1016/j.arr.2016.04.010 27125852

[6] Sardiello M, Palmieri M, di Ronza A, Medina DL, Valenza M, Gennarino VA, et al A gene network regulating lysosomal biogenesis and function. Science. 2009;325(5939):473–7. 10.1126/science.1174447 19556463

[7] Settembre C, Di Malta C, Polito VA, Garcia Arencibia M, Vetrini F, Erdin S, et al TFEB links autophagy to lysosomal biogenesis. Science. 2011;332(6036):1429–33. 10.1126/science.1204592 21617040 PMC3638014

[8] Cortes CJ, La Spada AR. TFEB dysregulation as a driver of autophagy dysfunction in neurodegenerative disease: Molecular mechanisms, cellular processes, and emerging therapeutic opportunities. Neurobiology of disease. 2019;122:83–93. 10.1016/j.nbd.2018.05.012 29852219 PMC6291370

[9] Martini-Stoica H, Xu Y, Ballabio A, Zheng H. The Autophagy-Lysosomal Pathway in Neurodegeneration: A TFEB Perspective. Trends in neurosciences. 2016;39(4):221–34. 10.1016/j.tins.2016.02.002 26968346 PMC4928589

[10] Decressac M, Mattsson B, Weikop P, Lundblad M, Jakobsson J, Bjorklund A. TFEB-mediated autophagy rescues midbrain dopamine neurons from alpha-synuclein toxicity. Proceedings of the National Academy of Sciences of the United States of America. 2013;110(19):E1817–26. 10.1073/pnas.1305623110 23610405 PMC3651458

[11] Torra A, Parent A, Cuadros T, Rodriguez-Galvan B, Ruiz-Bronchal E, Ballabio A, et al Overexpression of TFEB Drives a Pleiotropic Neurotrophic Effect and Prevents Parkinson’s Disease-Related Neurodegeneration. Molecular therapy: the journal of the American Society of Gene Therapy. 2018;26(6):1552–67. 29628303 10.1016/j.ymthe.2018.02.022 PMC5986717

[12] Gotzl JK, Lang CM, Haass C, Capell A. Impaired protein degradation in FTLD and related disorders. Ageing research reviews. 2016. 10.1016/j.arr.2016.04.008 27166223

[13] Nixon RA. The role of autophagy in neurodegenerative disease. Nature medicine. 2013;19(8):983–97. 10.1038/nm.3232 23921753

[14] Wyss-Coray T. Ageing, neurodegeneration and brain rejuvenation. Nature. 2016;539(7628):180–6. 10.1038/nature20411 27830812 PMC5172605

[15] Neumann M, Sampathu DM, Kwong LK, Truax AC, Micsenyi MC, Chou TT, et al Ubiquitinated TDP-43 in frontotemporal lobar degeneration and amyotrophic lateral sclerosis. Science. 2006;314(5796):130–3. 10.1126/science.1134108 17023659

[16] Baker M, Mackenzie IR, Pickering-Brown SM, Gass J, Rademakers R, Lindholm C, et al Mutations in progranulin cause tau-negative frontotemporal dementia linked to chromosome 17. Nature. 2006;442(7105):916–9. 10.1038/nature05016 16862116

[17] Cruts M, Gijselinck I, van der Zee J, Engelborghs S, Wils H, Pirici D, et al Null mutations in progranulin cause ubiquitin-positive frontotemporal dementia linked to chromosome 17q21. Nature. 2006;442(7501):920–4. 16862115 10.1038/nature05017

[18] Smith KR, Damiano J, Franceschetti S, Carpenter S, Canafoglia L, Morbin M, et al Strikingly different clinicopathological phenotypes determined by progranulin-mutation dosage. Am J Hum Genet. 2012;90(6):1102–7. 10.1016/j.ajhg.2012.04.021 22608501 PMC3370276

[19] Hu F, Padukkavidana T, Vægter CB, Brady OA, Zheng Y, Mackenzie IR, et al Sortilin-Mediated Endocytosis Determines Levels of the Frontotemporal Dementia Protein, Progranulin. Neuron. 2010;68(4):654–67. 10.1016/j.neuron.2010.09.034 21092856 PMC2990962

[20] Tanaka Y, Matsuwaki T, Yamanouchi K, Nishihara M. Increased lysosomal biogenesis in activated microglia and exacerbated neuronal damage after traumatic brain injury in progranulin-deficient mice. Neuroscience. 2013;250:8–19. 10.1016/j.neuroscience.2013.06.049 23830905

[21] Zhou X, Sun L, Bastos de Oliveira F, Qi X, Brown WJ, Smolka MB, et al Prosaposin facilitates sortilin-independent lysosomal trafficking of progranulin. The Journal of cell biology. 2015;210(6):991–1002. 10.1083/jcb.201502029 26370502 PMC4576858

[22] Gowrishankar S, Yuan P, Wu Y, Schrag M, Paradise S, Grutzendler J, et al Massive accumulation of luminal protease-deficient axonal lysosomes at Alzheimer’s disease amyloid plaques. Proceedings of the National Academy of Sciences of the United States of America. 2015;112(28):E3699–708. 10.1073/pnas.1510329112 26124111 PMC4507205

[23] Tanaka Y, Suzuki G, Matsuwaki T, Hosokawa M, Serrano G, Beach TG, et al Progranulin regulates lysosomal function and biogenesis through acidification of lysosomes. Human molecular genetics. 2017. 10.1093/hmg/ddx011 28073925

[24] Evers BM, Rodriguez-Navas C, Tesla RJ, Prange-Kiel J, Wasser CR, Yoo KS, et al Lipidomic and Transcriptomic Basis of Lysosomal Dysfunction in Progranulin Deficiency. Cell reports. 2017;20(11):2565–74. 10.1016/j.celrep.2017.08.056 28903038 PMC5757843

[25] Beel S, Moisse M, Damme M, De Muynck L, Robberecht W, Van Den Bosch L, et al Progranulin functions as a cathepsin D chaperone to stimulate axonal outgrowth in vivo. Human molecular genetics. 2017;26(15):2850–63. 10.1093/hmg/ddx162 28453791 PMC5886064

[26] Plowman GD, Green JM, Neubauer MG, Buckley SD, McDonald VL, Todaro GJ, et al The epithelin precursor encodes two proteins with opposing activities on epithelial cell growth. The Journal of biological chemistry. 1992;267(18):13073–8. 1618805

[27] Kao AW, McKay A, Singh PP, Brunet A, Huang EJ. Progranulin, lysosomal regulation and neurodegenerative disease. Nature reviews Neuroscience. 2017. 10.1038/nrn.2017.36 PMC6040832 28435163

[28] Lavergne V, Taft RJ, Alewood PF. Cysteine-rich mini-proteins in human biology. Curr Top Med Chem. 2012;12(14):1514–33. 22827521 10.2174/156802612802652411

[29] Palfree RG, Bennett HP, Bateman A. The Evolution of the Secreted Regulatory Protein Progranulin. PloS one. 2015;10(8):e0133749 10.1371/journal.pone.0133749 26248158 PMC4527844

[30] Cenik B, Sephton CF, Kutluk Cenik B, Herz J, Yu G. Progranulin: a proteolytically processed protein at the crossroads of inflammation and neurodegeneration. The Journal of biological chemistry. 2012;287(39):32298–306. 10.1074/jbc.R112.399170 22859297 PMC3463300

[31] Bateman A, Bennett HP. The granulin gene family: from cancer to dementia. Bioessays. 2009;31(11):1245–54. 10.1002/bies.200900086 19795409

[32] Holler CJ, Taylor G, Deng Q, Kukar T. Intracellular Proteolysis of Progranulin Generates Stable, Lysosomal Granulins that Are Haploinsufficient in Patients with Frontotemporal Dementia Caused by GRN Mutations. eNeuro. 2017;4(4). 10.1523/ENEURO.0100-17.2017 PMC5562298 28828399

[33] Lee CW, Stankowski JN, Chew J, Cook CN, Lam YW, Almeida S, et al The lysosomal protein cathepsin L is a progranulin protease. Molecular neurodegeneration. 2017;12(1):55 10.1186/s13024-017-0196-6 28743268 PMC5526245

[34] Zhou X, Paushter DH, Feng T, Sun L, Reinheckel T, Hu F. Lysosomal processing of progranulin. Molecular neurodegeneration. 2017;12(1):62 10.1186/s13024-017-0205-9 28835281 PMC5569495

[35] Tolkatchev D, Malik S, Vinogradova A, Wang P, Chen Z, Xu P, et al Structure dissection of human progranulin identifies well-folded granulin/epithelin modules with unique functional activities. Protein science: a publication of the Protein Society. 2008;17(4):711–24. 18359860 10.1110/ps.073295308 PMC2271164

[36] He Z, Bateman A. Progranulin (granulin-epithelin precursor, PC-cell-derived growth factor, acrogranin) mediates tissue repair and tumorigenesis. J Mol Med. 2003;81(10):600–12. 10.1007/s00109-003-0474-3 12928786

[37] Kessenbrock K, Frohlich L, Sixt M, Lammermann T, Pfister H, Bateman A, et al Proteinase 3 and neutrophil elastase enhance inflammation in mice by inactivating antiinflammatory progranulin. J Clin Invest. 2008;118(7):2438–47. 10.1172/JCI34694 18568075 PMC2430496

[38] Zhu J, Nathan C, Jin W, Sim D, Ashcroft GS, Wahl SM, et al Conversion of proepithelin to epithelins: roles of SLPI and elastase in host defense and wound repair. Cell. 2002;111(6):867–78. 10.1016/s0092-8674(02)01141-8 12526812

[39] Salazar N, Butler V, Argouarch A, Nakamura A, Mason A, McCurdy H, et al The progranulin cleavage products, granulins, exacerbate TDP-43 toxicity and increase TDP-43 levels. J Neurosci. 2015;35(25):9315–28. 10.1523/JNEUROSCI.4808-14.2015 26109656 PMC4478251

[40] Judy ME, Nakamura A, Huang A, Grant H, McCurdy H, Weiberth KF, et al A shift to organismal stress resistance in programmed cell death mutants. PLoS genetics. 2013;9(9):e1003714 10.1371/journal.pgen.1003714 24068943 PMC3778000

[41] Heschl MF, Baillie DL. Characterization of the hsp70 multigene family of Caenorhabditis elegans. DNA. 1989;8(4):233–43. 2766926 10.1089/dna.1.1989.8.233

[42] Calfon M, Zeng H, Urano F, Till JH, Hubbard SR, Harding HP, et al IRE1 couples endoplasmic reticulum load to secretory capacity by processing the XBP-1 mRNA. Nature. 2002;415(6867):92–6. 10.1038/415092a 11780124

[43] Kauffman AL, Ashraf JM, Corces-Zimmerman MR, Landis JN, Murphy CT. Insulin signaling and dietary restriction differentially influence the decline of learning and memory with age. PLoS biology. 2010;8(5):e1000372 10.1371/journal.pbio.1000372 20502519 PMC2872642

[44] Torayama I, Ishihara T, Katsura I. Caenorhabditis elegans integrates the signals of butanone and food to enhance chemotaxis to butanone. The Journal of neuroscience: the official journal of the Society for Neuroscience. 2007;27(4):741–50. 10.1523/JNEUROSCI.4312-06.2007 PMC6672901 17251413

[45] Kao AW, Eisenhut RJ, Herl Martens L, Nakamura A, Huang A, Bagley JA, et al A neurodegenerative disease mutation that accelerates the clearance of apoptotic cells. Proceedings of the National Academy of Sciences of the United States of America. 2011;108(11):4441–6. 10.1073/pnas.1100650108 21368173 PMC3060230

[46] Petkau TL, Neal SJ, Orban PC, MacDonald JL, Hill AM, Lu G, et al Progranulin expression in the developing and adult murine brain. J Comp Neurol. 2010;518(19):3931–47. 10.1002/cne.22430 20737593

[47] Petkau TL, Leavitt BR. Progranulin in neurodegenerative disease. Trends in neurosciences. 2014;37(7):388–98. 10.1016/j.tins.2014.04.003 24800652

[48] Holler CJ, Taylor G, McEachin ZT, Deng Q, Watkins WJ, Hudson K, et al Trehalose upregulates progranulin expression in human and mouse models of GRN haploinsufficiency: a novel therapeutic lead to treat frontotemporal dementia. Molecular neurodegeneration. 2016;11(1):46 10.1186/s13024-016-0114-3 27341800 PMC4919863

[49] Fares H, Greenwald I. Genetic analysis of endocytosis in Caenorhabditis elegans: coelomocyte uptake defective mutants. Genetics. 2001;159(1):133–45. 11560892 10.1093/genetics/159.1.133 PMC1461804

[50] Lapierre LR, De Magalhaes Filho CD, McQuary PR, Chu CC, Visvikis O, Chang JT, et al The TFEB orthologue HLH-30 regulates autophagy and modulates longevity in Caenorhabditis elegans. Nature communications. 2013;4:2267 10.1038/ncomms3267 23925298 PMC3866206

[51] Settembre C, Zoncu R, Medina DL, Vetrini F, Erdin S, Erdin S, et al A lysosome-to-nucleus signalling mechanism senses and regulates the lysosome via mTOR and TFEB. The EMBO journal. 2012;31(5):1095–108. 10.1038/emboj.2012.32 22343943 PMC3298007

[52] Luckett S, Garcia RS, Barker JJ, Konarev AV, Shewry PR, Clarke AR, et al High-resolution structure of a potent, cyclic proteinase inhibitor from sunflower seeds. Journal of molecular biology. 1999;290(2):525–33. 10.1006/jmbi.1999.2891 10390350

[53] Trivedi MV, Laurence JS, Siahaan TJ. The role of thiols and disulfides on protein stability. Current protein & peptide science. 2009;10(6):614–25. 19538140 10.2174/138920309789630534 PMC3319691

[54] Yamada K, Matsushima R, Nishimura M, Hara-Nishimura I. A slow maturation of a cysteine protease with a granulin domain in the vacuoles of senescing Arabidopsis leaves. Plant physiology. 2001;127(4):1626–34. 11743107 PMC133567

[55] Pennacchio LA, Lehesjoki AE, Stone NE, Willour VL, Virtaneva K, Miao J, et al Mutations in the gene encoding cystatin B in progressive myoclonus epilepsy (EPM1). Science. 1996;271(5256):1731–4. 10.1126/science.271.5256.1731 8596935

[56] Butler VJ, Cortopassi WA, Argouarch AR, Ivry SL, Craik CS, Jacobson MP, et al Progranulin Stimulates the In Vitro Maturation of Pro-Cathepsin D at Acidic pH. Journal of molecular biology. 2019;431(5):1038–47. 10.1016/j.jmb.2019.01.027 30690031 PMC6613950

[57] Valdez C, Wong YC, Schwake M, Bu G, Wszolek ZK, Krainc D. Progranulin-mediated deficiency of cathepsin D results in FTD and NCL-like phenotypes in neurons derived from FTD patients. Human molecular genetics. 2017. 10.1093/hmg/ddx364 PMC5886207 29036611

[58] Zhou X, Paushter DH, Feng T, Pardon CM, Mendoza CS, Hu F. Regulation of cathepsin D activity by the FTLD protein progranulin. Acta neuropathologica. 2017;134(1):151–3. 10.1007/s00401-017-1719-5 28493053 PMC5568051

[59] Lui H, Zhang J, Makinson SR, Cahill MK, Kelley KW, Huang HY, et al Progranulin Deficiency Promotes Circuit-Specific Synaptic Pruning by Microglia via Complement Activation. Cell. 2016;165(4):921–35. 10.1016/j.cell.2016.04.001 27114033 PMC4860138

[60] Van Kampen JM, Baranowski D, Kay DG. Progranulin gene delivery protects dopaminergic neurons in a mouse model of Parkinson’s disease. PloS one. 2014;9(5):e97032 10.1371/journal.pone.0097032 24804730 PMC4013129

[61] Minami SS, Min SW, Krabbe G, Wang C, Zhou Y, Asgarov R, et al Progranulin protects against amyloid beta deposition and toxicity in Alzheimer’s disease mouse models. Nature medicine. 2014;20(10):1157–64. 10.1038/nm.3672 25261995 PMC4196723

[62] Alberici A, Archetti S, Pilotto A, Premi E, Cosseddu M, Bianchetti A, et al Results from a pilot study on amiodarone administration in monogenic frontotemporal dementia with granulin mutation. Neurological sciences: official journal of the Italian Neurological Society and of the Italian Society of Clinical Neurophysiology. 2014;35(8):1215–9. 10.1007/s10072-014-1683-y 24569924

[63] Cenik B, Sephton CF, Dewey CM, Xian X, Wei S, Yu K, et al Suberoylanilide hydroxamic acid (vorinostat) up-regulates progranulin transcription: rational therapeutic approach to frontotemporal dementia. The Journal of biological chemistry. 2011;286(18):16101–8. 10.1074/jbc.M110.193433 21454553 PMC3091219

[64] Sha SJ, Miller ZA, Min SW, Zhou Y, Brown J, Mitic LL, et al An 8-week, open-label, dose-finding study of nimodipine for the treatment of progranulin insufficiency from GRN gene mutations. Alzheimer’s & dementia. 2017;3(4):507–12. 10.1016/j.trci.2017.08.002 PMC5671622 29124108

[65] She A, Kurtser I, Reis SA, Hennig K, Lai J, Lang A, et al Selectivity and Kinetic Requirements of HDAC Inhibitors as Progranulin Enhancers for Treating Frontotemporal Dementia. Cell Chem Biol. 2017;24(7):892–906 e5. 10.1016/j.chembiol.2017.06.010 28712747 PMC5695697

[66] Amado DA, Rieders JM, Diatta F, Hernandez-Con P, Singer A, Mak JT, et al AAV-Mediated Progranulin Delivery to a Mouse Model of Progranulin Deficiency Causes T Cell-Mediated Toxicity. Molecular therapy: the journal of the American Society of Gene Therapy. 2019;27(2):465–78. 30559071 10.1016/j.ymthe.2018.11.013 PMC6369714

[67] Brenner S. The genetics of Caenorhabditis elegans. Genetics. 1974;77(1):71–94. 4366476 10.1093/genetics/77.1.71 PMC1213120

[68] Cuervo AM, Dice JF, Knecht E. A population of rat liver lysosomes responsible for the selective uptake and degradation of cytosolic proteins. The Journal of biological chemistry. 1997;272(9):5606–15. 10.1074/jbc.272.9.5606 9038169

[69] Katoh K, Rozewicki J, Yamada KD. MAFFT online service: multiple sequence alignment, interactive sequence choice and visualization. Brief Bioinform. 2017. 10.1093/bib/bbx108 PMC6781576 28968734

[70] Madeira F, Park YM, Lee J, Buso N, Gur T, Madhusoodanan N, et al The EMBL-EBI search and sequence analysis tools APIs in 2019. Nucleic acids research. 2019. 10.1093/nar/gkz268 PMC6602479 30976793

[71] Butler VJ, Cortopassi WA, Gururaj S, Wang AL, Pierce OM, Jacobson MP, et al Multi-Granulin Domain Peptides Bind to Pro-Cathepsin D and Stimulate Its Enzymatic Activity More Effectively Than Progranulin in Vitro. Biochemistry. 2019. 10.1021/acs.biochem.9b00275 PMC6666309 31099551

[72] Olsson MH, Sondergaard CR, Rostkowski M, Jensen JH. PROPKA3: Consistent Treatment of Internal and Surface Residues in Empirical pKa Predictions. Journal of chemical theory and computation. 2011;7(2):525–37. 10.1021/ct100578z 26596171

[73] Dobin A, Davis CA, Schlesinger F, Drenkow J, Zaleski C, Jha S, et al STAR: ultrafast universal RNA-seq aligner. Bioinformatics. 2013;29(1):15–21. 10.1093/bioinformatics/bts635 23104886 PMC3530905

[74] Robinson MD, McCarthy DJ, Smyth GK. edgeR: a Bioconductor package for differential expression analysis of digital gene expression data. Bioinformatics. 2010;26(1):139–40. 10.1093/bioinformatics/btp616 19910308 PMC2796818

[75] Law CW, Chen Y, Shi W, Smyth GK. voom: Precision weights unlock linear model analysis tools for RNA-seq read counts. Genome Biol. 2014;15(2):R29 10.1186/gb-2014-15-2-r29 24485249 PMC4053721

[76] Eden E, Lipson D, Yogev S, Yakhini Z. Discovering motifs in ranked lists of DNA sequences. PLoS Comput Biol. 2007;3(3):e39 10.1371/journal.pcbi.0030039 17381235 PMC1829477

[77] Eden E, Navon R, Steinfeld I, Lipson D, Yakhini Z. GOrilla: a tool for discovery and visualization of enriched GO terms in ranked gene lists. BMC Bioinformatics. 2009;10:48 10.1186/1471-2105-10-48 19192299 PMC2644678

