# Supplementary figures and images for: Age- and stress-associated C. elegans granulins impair lysosomal function and induce a compensatory HLH-30/TFEB transcriptional response
Source: PLoS Genet. 2019 Aug 9;15(8):e1008295. doi: 10.1371/journal.pgen.1008295 (PMC6703691; doi:10.1371/journal.pgen.1008295)

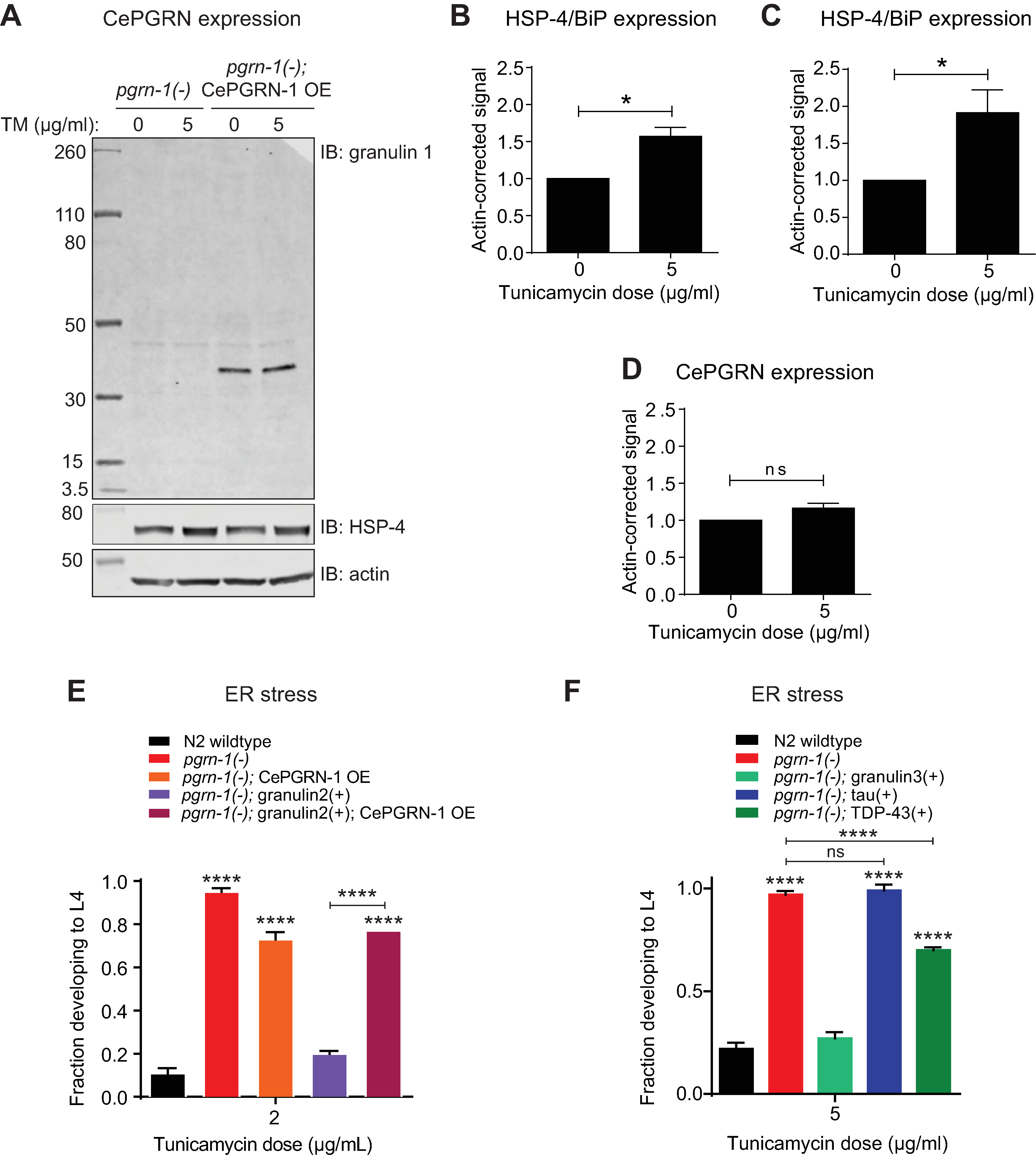

Supplement: S1 Fig — (A) Total worm lysates from synchronized day 1 adult animals over-expressing C. elegans progranulin (CePGRN-1 OE) were immunoblotted with an anti-granulin 1 antibody after being grown in the presence and absence of 5μg tunicamycin (3 biological replicates, TM = tunicamycin). Anti-HSP-4/BiP, the nematode homolog of human BiP/Grp78, was used to confirm the induction of ER stress and anti-actin was used as a loading control. (B) Quantification of HSP-4/BiP expression in the presence and absence of 5μg tunicamycin in pgrn-1(-) animals (3 biological replicates, error bars show mean ± SEM). (C) Quantification of HSP-4/BiP expression in the presence and absence of 5μg tunicamycin in animals with C. elegans progranulin over-expression (3 biological replicates, error bars show mean ± SEM). (D) Quantification of CePGRN-1 expression in the presence and absence of 5μg tunicamycin in animals with C. elegans progranulin OE (3 biological replicates, error bars show mean ± SEM). (E) Wild-type (N2) and pgrn-1(-) animals with and without C. elegans granulin 2 and progranulin over-expression were subjected to ER stress with tunicamycin (2 μg / ml). The fraction developing to L4 stage was quantified (n = 50, 3 biological replicates). Granulin 1 and 3 could not be tested because these transgenes are on the same chromosome as the progranulin over-expression transgene and recombinants were not obtained from the crosses. (F) Wild-type (N2) and pgrn-1(-) animals with and without C. elegans granulin 3, human 1N4R tau and human TDP-43 over-expression were subjected to ER stress with tunicamycin (5 μg / ml). The fraction developing to L4 stage was quantified (n = 50, 3 biological replicates). (TIF) [file pgen.1008295.s001.tif]

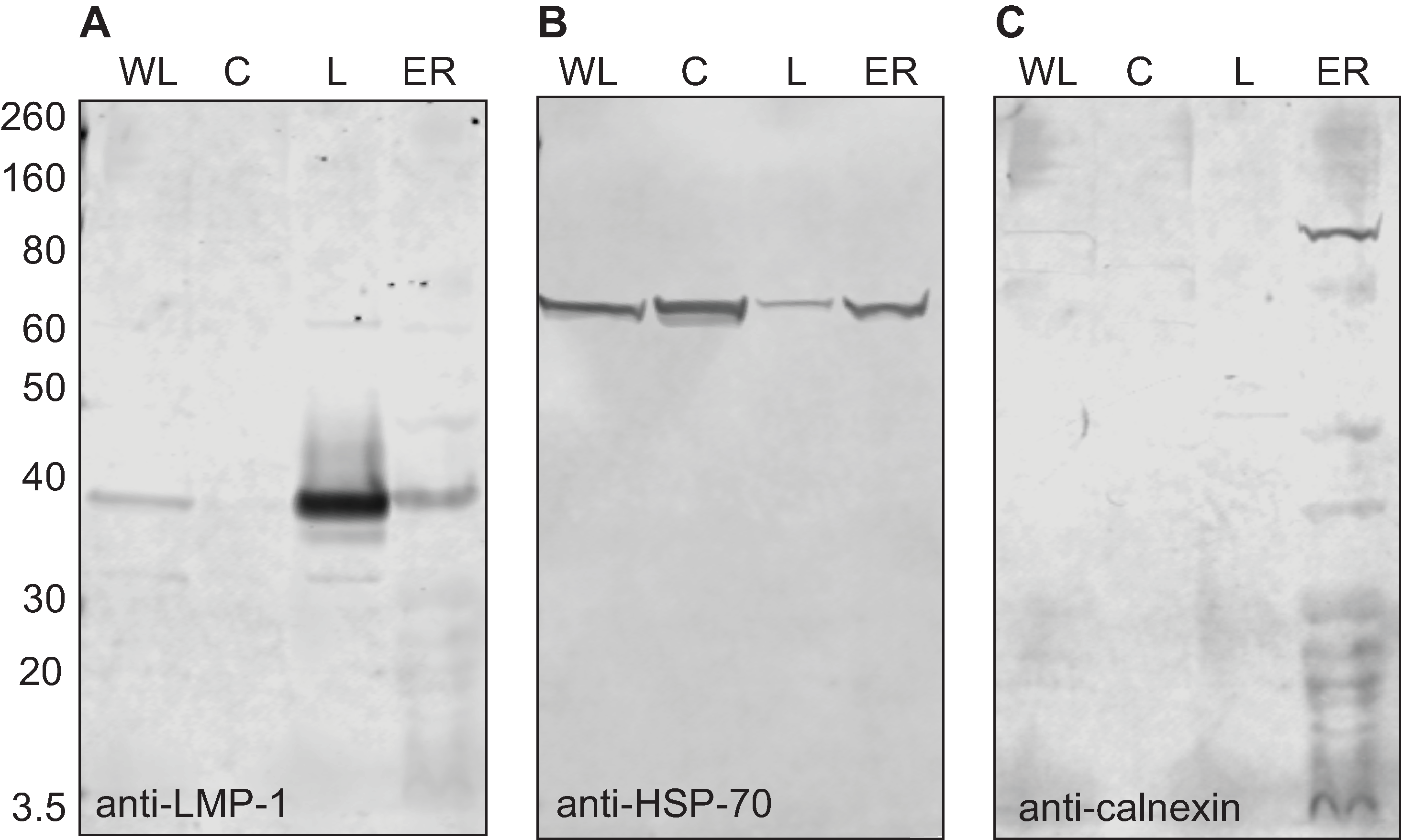

Supplement: S2 Fig — (A-C) Validation of subcellular fractions from C. elegans by blotting for fraction-specific markers: (A) anti-LMP-1 is specific for the lysosomal fraction, (B) anti-HSP-70 which is normally found in both cytosolic and lysosomal fractions, and (C) calnexin which localizes to the ER fraction. Similar results were observed in three independent Western blots. WL = whole lysate, C = cytosol, L = lysosomes, ER = endoplasmic reticulum. (TIF) [file pgen.1008295.s002.tif]

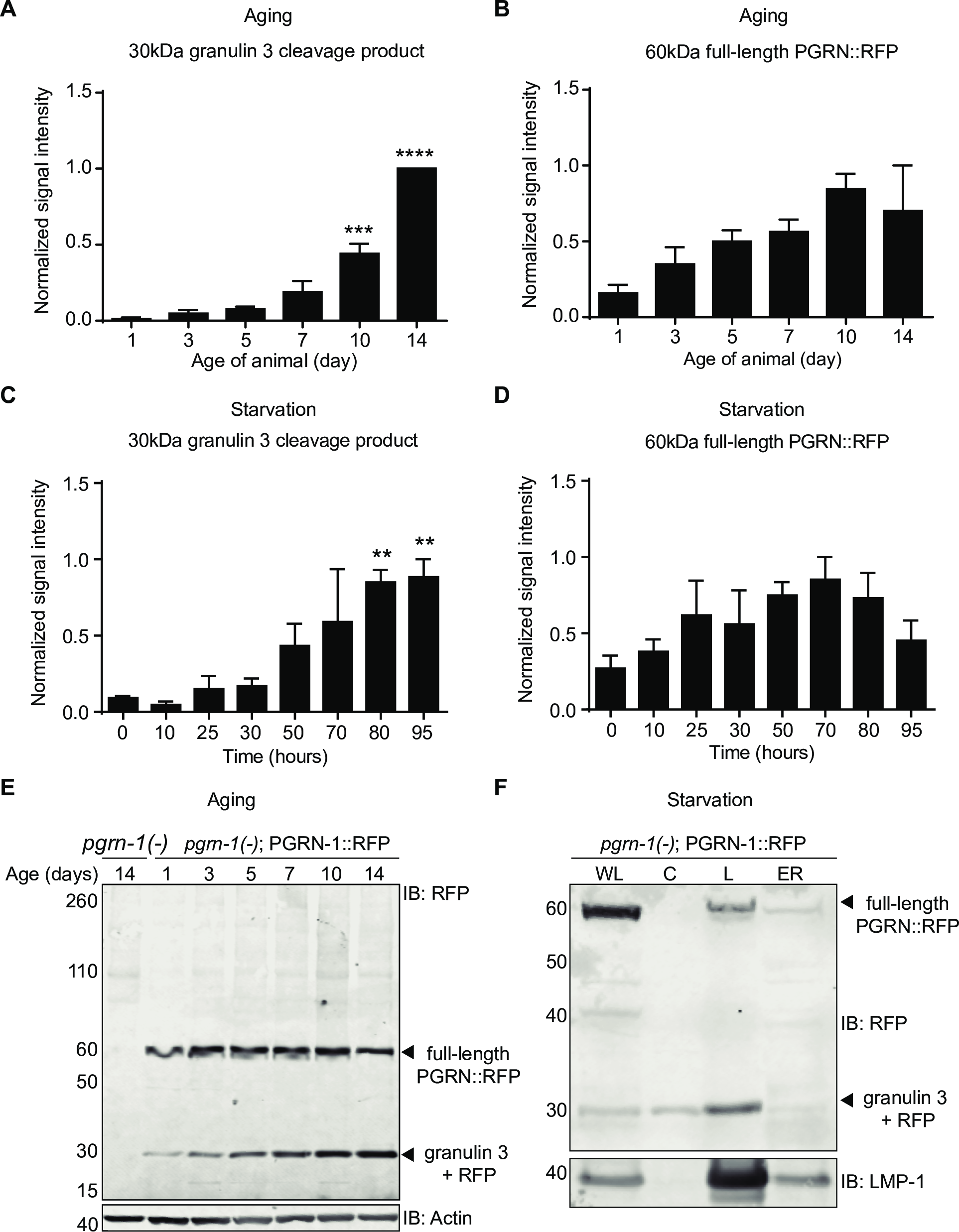

Supplement: S3 Fig — (A-D) Quantification of actin-corrected mean band intensities from Western blot of C. elegans PGRN-1::RFP lysates with (A-B) aging, and (C-D) starvation. Actin-corrected mean band intensities were normalized to highest value per experiment (data from three biological replicates is shown, values shown are mean ± SEM, one-way ANOVA and Tukey multiple comparisons test, **P<0.01, ***P<0.001, ****P<0.0001). (E) In C. elegans pgrn-1(-); PGRN-1::RFP lysates the most prominent cleavage product at ~30 kDa was recognized by both granulin 3 and RFP antibodies. (F) Subcellular fractionation of pgrn-1(-); PGRN::RFP animals. Whole lysate (WL), cytosol (C), lysosome (L) and endoplasmic reticulum (ER) fractions from starved (70 hours off-food) animals were immunoblotted with anti-RFP and anti-LMP-1 antibodies. (TIF) [file pgen.1008295.s003.tif]

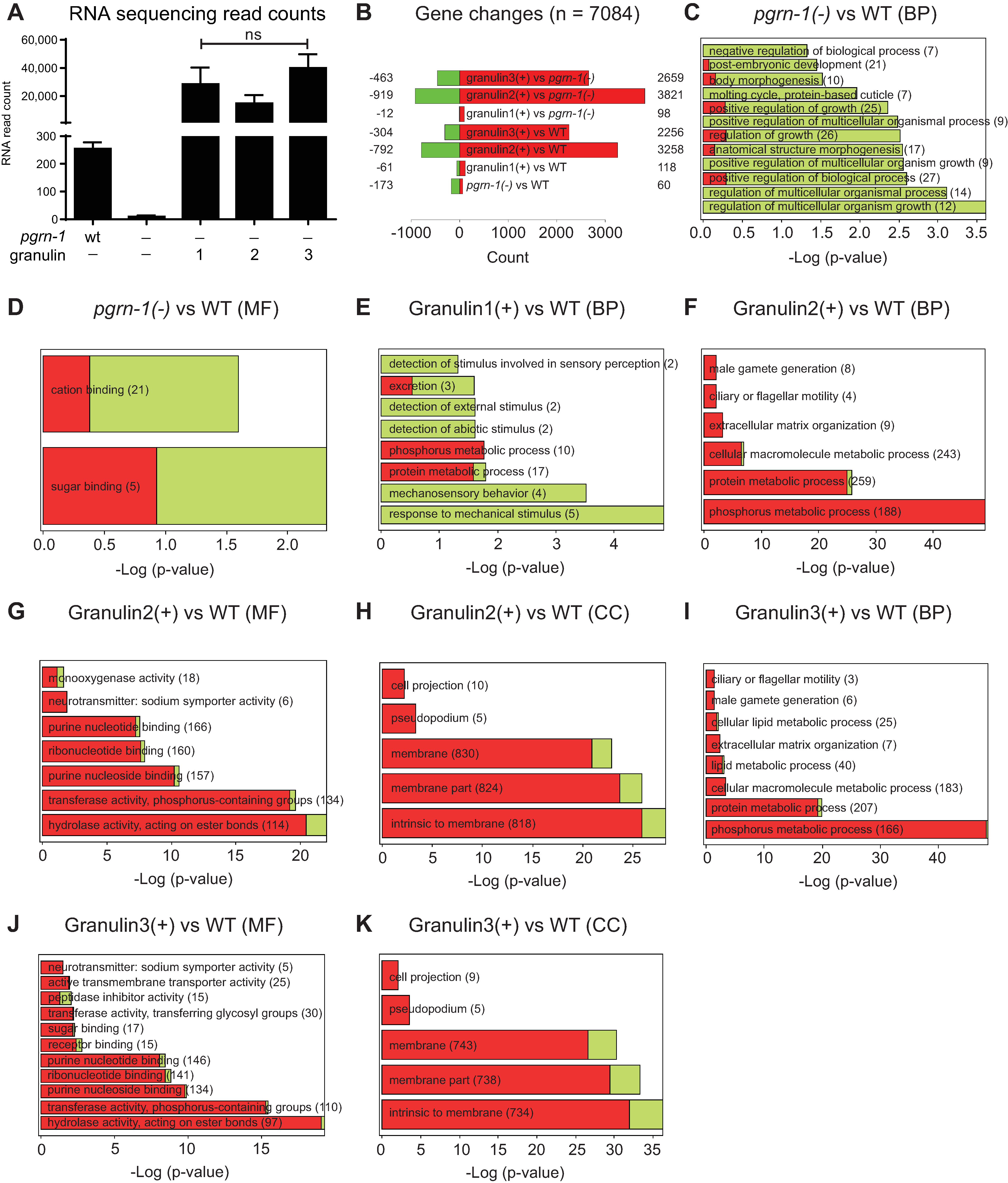

Supplement: S4 Fig — (A) RNA sequencing read counts at the progranulin gene locus (n = 4). Error bars show the mean ± SEM, one-way ANOVA with post-hoc Tukey multiple comparisons test. (B) Numbers of differentially expressed genes (DEGs) identified in the comparisons indicated. (C-D) GO term enrichment analysis for pgrn-1(-) animals compared to wildtype (WT) for (C) Biological Process (BP) and (D) Molecular Function (MF) categories. (E) GO term enrichment analysis for pgrn-1(-); granulin 1(+) animals compared to WT for BP. (F-H) GO term enrichment analysis for pgrn-1(-); granulin 2(+) animals compared to WT for (F) BP, (G) MF and (H) Cellular Component (CC) categories. (I-K) GO term enrichment analysis for pgrn-1(-); granulin 3(+) animals compared to WT for (I) BP, (J) MF and (K) CC categories. For all panels, data from four independent biological replicates are shown (except for granulin 1 where one sample was excluded as a quality control outlier). The significance cut-off was a false discovery rate (FDR) of P<0.05, up-regulated = red, down-regulated = green. For C-K, the number of DEGs identified within each GO term is indicated in parentheses. (TIF) [file pgen.1008295.s004.tif]

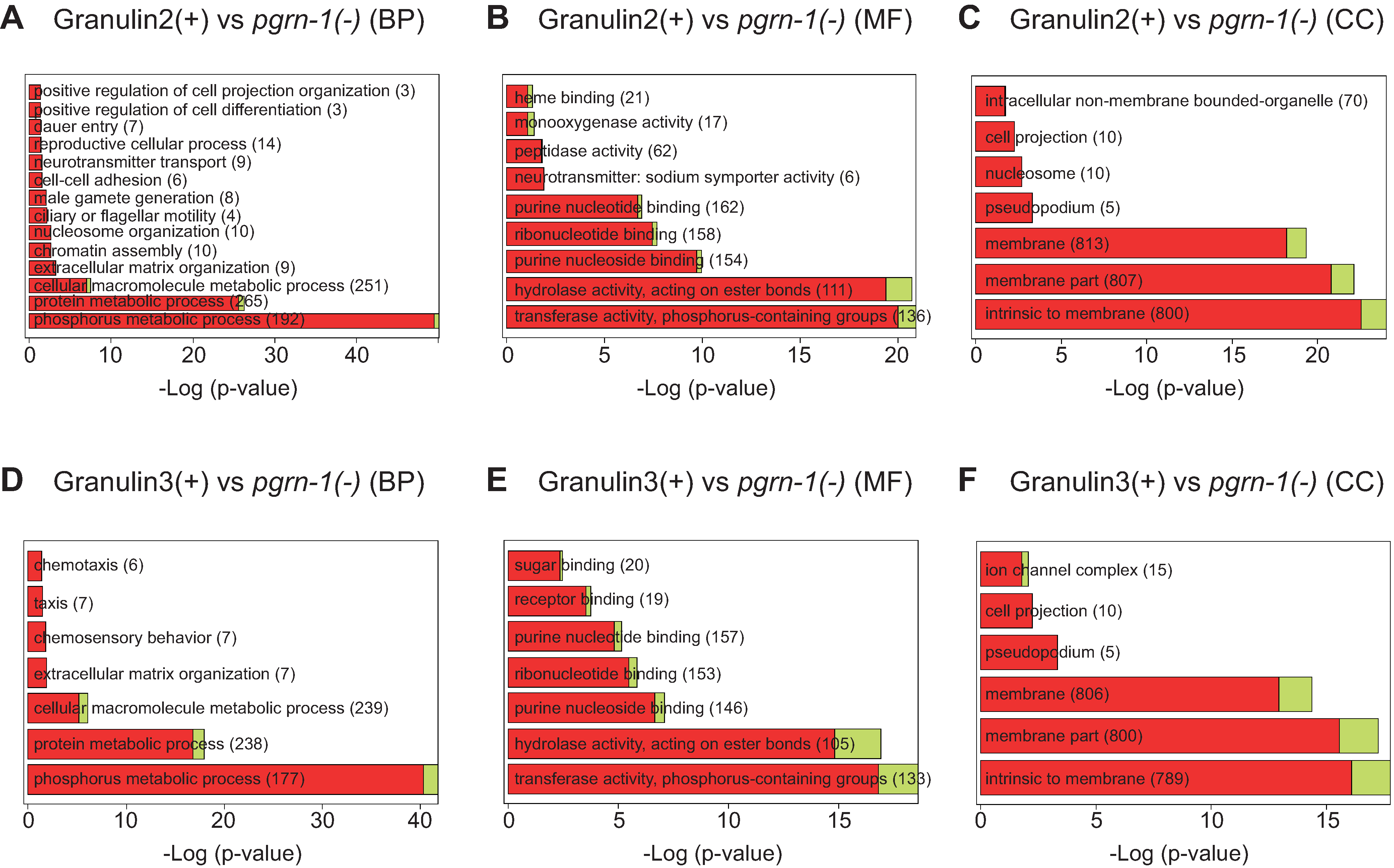

Supplement: S5 Fig — (A-C) GO term enrichment analysis for pgrn-1(-); granulin 2(+) animals compared to pgrn-1(-) for (A) Biological Process (BP), (B) Molecular Function (MF) and (C) Cellular Component (CC) categories. (D-F) GO term enrichment analysis for pgrn-1(-); granulin 3(+) animals compared to WT for (D) BP and (E) MF and (F) CC categories. For all panels, data from four independent biological replicates are shown. The significance cut-off was a false discovery rate (FDR) of P<0.05, up-regulated = red, down-regulated = green. The number of DEGs identified within each GO term is indicated in parentheses. (TIF) [file pgen.1008295.s005.tif]

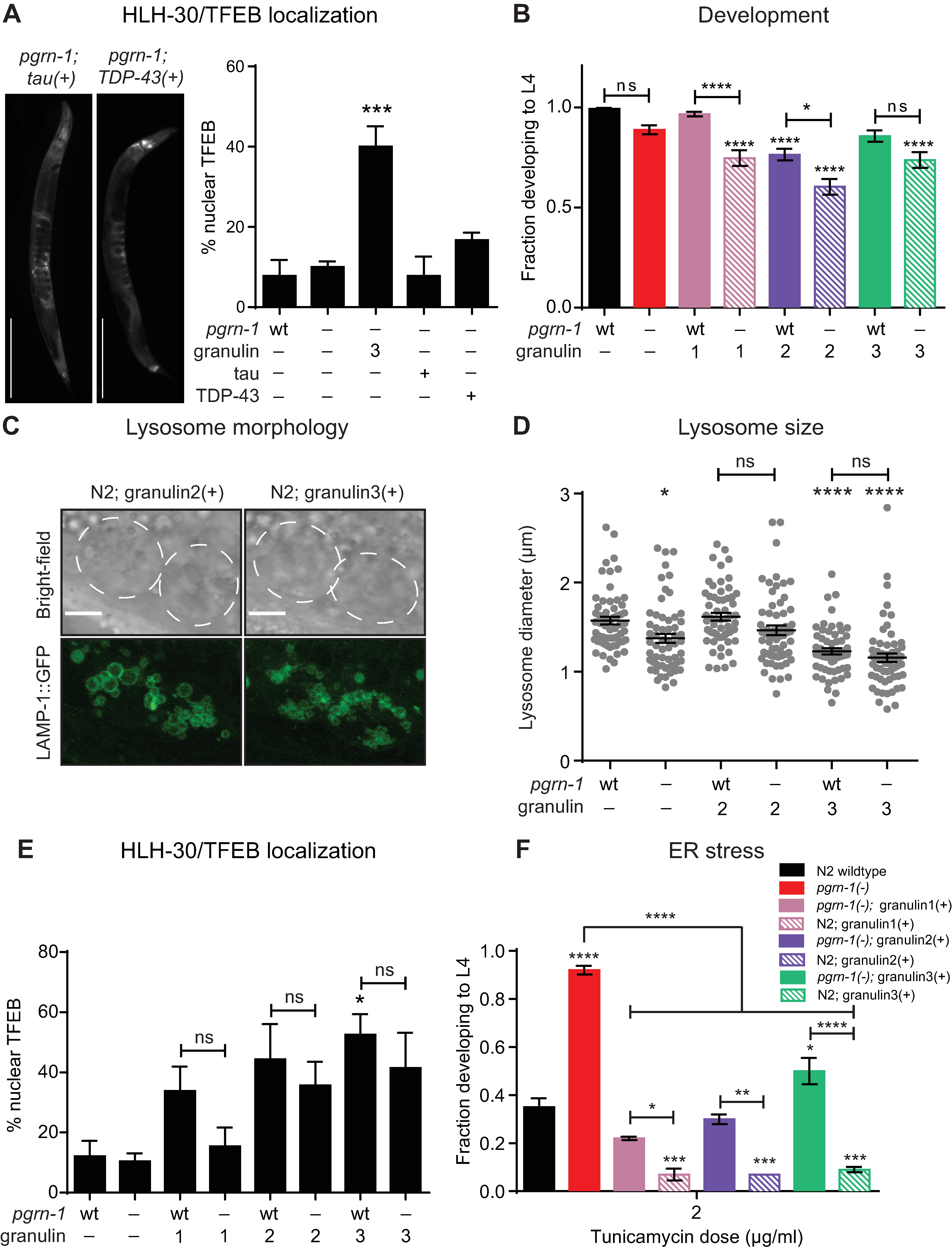

Supplement: S6 Fig — (A) Percentage of animals with nuclear localized HLH-30::GFP (n = 90 animals from 3 biological replicates). Representative images of pgrn-1(-); tau(+) and pgrn-1(-); TDP-43(+) animals expressing HLH-30::GFP are shown (scale bar = 200 μm). (B) Wild-type (N2) and pgrn-1(-) animals with and without granulin expression were staged as embryos. Animals were scored for development to L4 stage (n = 50, 3 biological replicates). (C) Representative light and fluorescent confocal images of anterior coelomocyte cells expressing LMP-1::GFP in N2; granulin 2(+) and N2; granulin 3(+) animals. Scale bars are 10 μm. Dashed white lines mark the outline of each coelomocyte cell. (D) Lysosomal diameter measurements from anterior coelomocyte cells (n = 60). Mean values (μm): wt: 1.57 ± 0.04, pgrn-1(-): 1.37 ± 0.05, N2; granulin 2(+): 1.62 ± 0.04, pgrn-1(-); granulin2(+): 1.47 ± 0.05, N2; granulin 3(+): 1.23 ± 0.03, pgrn-1(-); granulin3(+): 1.16 ± 0.05. (E) Percentage of animals with nuclear localized HLH-30::GFP (n = 120 animals from 4 biological replicates). (F) Wild-type (N2) and pgrn-1(-) animals with and without granulin expression were subjected to ER stress with tunicamycin (5 μg / ml). The fraction developing to L4 stage was quantified (n = 50, 3 biological replicates). For panels A, B, C, E and F, error bars show the mean ± SEM, one-way ANOVA with post-hoc Tukey multiple comparisons test. Comparisons are to wildtype unless otherwise indicated (*P<0.05, **P<0.01, ***P<0.001, ****P<0.0001, wt = wildtype, ns = not significant). (TIF) [file pgen.1008295.s006.tif]
